# The collaborative mode by *PmSVPs* and *PmDAMs* reveals neofunctionalization in the switch of the flower bud development and dormancy for *Prunus mume*

**DOI:** 10.3389/fpls.2022.1023628

**Published:** 2022-12-06

**Authors:** Kai Zhao, Yuzhen Zhou, Yan Zheng, Rui-yue Zheng, Meijuan Hu, Yan Tong, Xianmei Luo, Yangting Zhang, Ming-li Shen

**Affiliations:** ^1^ College of Life Sciences, Fujian Normal University, Fuzhou, China; ^2^ College of Landscape Architecture, Ornamental Plant Germplasm Resources Innovation and Engineering Application Research Center at College of Landscape Architecture, Key Laboratory of National Forestry and Grassland Administration for Orchid Conservation and Utilization at College of Landscape Architecture, Fujian Agriculture and Forestry University, Fuzhou, China

**Keywords:** *Prunus mume*, SVP gene, DAM gene, floral organ development, dormancy, yeast two-hybrid assays

## Abstract

*Prunus mume* (Rosaceae, Prunoideae) serves as an excellent ornamental woody plant with a large-temperature-range cultivation scope. Its flower buds require a certain low temperature to achieve flowering circulation. Thus, it is important to delve into the processes of flower bud differentiation and dormancy, which affected its continuous flowering. These processes are generally considered as regulation by the MADS-box homologs, *SHORT VEGETATIVE PHASE* (*SVP*), and *DORMANCY-ASSOCIATED MADS-BOX* (*DAM*). However, a precise model on their interdependence and specific function, when acting as a complex in the flower development of *P. mume*, is needed. Therefore, this study highlighted the integral roles of *PmDAMs* and *PmSVPs* in flower organ development and dormancy cycle. The segregation of *PmDAMs* and *PmSVPs* in a different cluster suggested distinct functions and neofunctionalization. The expression pattern and yeast two-hybrid assays jointly revealed that eight genes were involved in the floral organ development stages, with *PmDAM1* and *PmDAM5* specifically related to prolificated flower formation. *PmSVP1*–*2* mingled in the protein complex in bud dormancy stages with *PmDAMs*. Finally, we proposed the hypothesis that PmSVP1 and PmSVP2 could combine with PmDAM1 to have an effect on flower organogenesis and interact with PmDAM5 and PmDAM6 to regulate flower bud dormancy. These findings could help expand the current molecular mechanism based on MADS-box genes during flower bud development and dormancy.

## Introduction

Functional plants always change our routine life in a particular way, people are willing to unfold the mechanism, and a better usage can be applied to them. One significant part is that the modern floriculture industry calls for continuous flowering. *Prunus mume* (Mei flower) has been grown as woody flowers with over 3,000 years of cultivation history in China. After long-term natural and artificial selection in current breeding, we can enjoy cultivars with full aroma, different petal colors, and different branch types (Zhang et al., 2018). However, as representative woody plants in Rosaceae, Mei flowers experienced seasonal flowering (Zhang et al., 2012); the continuous flowering composed by floral ontogeny, organ differentiation, and bud expansion was stopped during cold-weather days. A defense mechanism is set up to protect the plant from cold damage, called dormancy. In previous research, we found the key roles of DAM genes and raised the complex model (Zhao et al., 2018a). As per our further observation, flower bud differentiation associated with flower shapes was completed in autumn; then, all flower buds stop growing at a certain low temperature. Naturally, it could get a quick release from dormancy and bloom early in spring. These processes which impact the ornamental value of *P. mume* are supposed to be regulated by the MADS-box gene family. Beyond this, a crosstalk of *PmCBF*s and *PmDAM*s on phytohormones was recorded, which highlight the inverse function of gibberellic acid (GA) and abscisic acid in this process (Zhao et al., 2018b). Some studies have shown that two *PmSVPs* and six *PmDAMs* belong to the MADS-box gene family, and more members were identified in one to two clades and characterized in *P. mume* and species in Rosaceae (Falavigna et al., 2018). Fine transcriptomes of *P. mume* analysis confirm the core co-expression relations to dormancy (Li et al., 2021). Despite the fact that these genes play vital roles in the floral organ development and dormancy cycle (Sasaki et al., 2011), little is known on the functional and structural associations between *PmSVPs* and *PmDAMs.*

*SVP* genes, which belongs to the STMADS11 subfamily of MADS-box gene family, are a crucial flowering repressor in *Arabidopsis thaliana* (Blazquez and Weigel, 2000). A homologous gene of *AtSVP* with similar functions has been identified in different herbaceous plants, such as *Brassica pekinensis* (Lee et al., 2000) and *Narcissus tazetta* (Li et al., 2015). Moreover, SVP genes were regarded as significantly expanding in Rosaceae, as there are about four times the number of members than those in Brassicaceae (Liu et al., 2020). It implied a more complicated and sensitive control in dormancy. *SVP* genes are implicated in floral transition and may affect the identity of the floral meristem (Liu et al., 2018). In perennial species, several reports evaluated the function of *DAM* and *SVP*-like genes in heterologous systems. In *Eucalyptus grandis*, the overexpression of *SVP*-like gene (*EgrSVP*) resulted in delaying the flowering time and increasing the inflorescence (Brill and Watson, 2004). The ectopic expression of *Poncirus trifoliata SVP* in wild-type *A. thaliana* induced some changes such as delayed flowering, additional trichomes, and floral defects (Li et al., 2010). In addition, *SVP*-like genes have been proposed to regulate dormancy. In *P. mume*, the overexpression of *PmSVP1* and *PmSVP2* in *A. thaliana* resulted in changes of the floral organs, additional trichomes, leaf-like sepals, and increased rosette branches (Li et al., 2017). Six tandemly duplicated *PmDAMs* share a sequence homology to *SVP* genes, which appeared to inherit functions from the current *PmSVPs*. Processing a growth inhibitory effect, the overexpression genes from sweet cherries and *Prunus mume* displayed a relevant delay of floral organofaction (Li et al., 2017; Wang et al., 2021). Beyond these discoveries, the current omics data of transcriptome and small RNA sequencing displayed the joint roles of SVPs and DAMs in *Prunus mume*. All members functioned by a network control in floral bud break and dormancy cycling (Zhang et al., 2022).

The *DAM* genes belong to the MIKC^C^ type of MADS-box gene. The MIKC^C^ type of genes could influence pollen development in *A. thaliana* (Liu et al., 2013). *PpeMADS20* and *PpeMADS36* are highly expressed in pollens and play a role in floral organ formation (Wells et al., 2015). The overexpression of *EeDAM1* from *Euphorbia esula* in *Arabidopsis* slightly delayed the flowering (Horvath et al., 2010). These studies could contribute to explore the role of *DAM* genes in regulating flower development. Furthermore, *DAM* genes were initially found in *Ever-growing* mutant of *P. persica* and well known as regulators associated with growth and dormancy cycle (Li et al., 2009). The expression of *DAM* genes of *P. avium* reached the highest spot during endodormancy, which is compatible with the role as repressors of endodormancy release or bud break (Rothkegel et al., 2017). By comparing the promoter regions of the *DAM* genes of *P. trichocarpa* and *E. esula*, it was found that some expression patterns are critical to the feedback of the stimulus factors that induce dormancy. Current inventions provided evidence that in pear alternative splicing of PpDAM1, especially *PpDAM1.2* showed a predominantly higher expression than *PpDAM1.1* and *PpDAM1.3* in two cultivars and thus could play a crucial role in the pear flower bud dormancy process (Li et al., 2021).

MADS-box proteins can widely form multimeric complexes with other proteins to regulate plant development. In *Arabidopsis*, SVP proteins form complexes with some MADS-box proteins to regulate flowering time and flower development. SVP and FLC proteins form a complex which delays flowering by inhibiting the expression of the floral integrator genes *FT* and *SOC1* as well as GA-related genes (Xie et al., 2021). PmDAM6, which interacts with PmSOC1, may participate in floral organogenesis, dormancy transition, and flowering time regulation in *P. mume* (Yong et al., 2021). Nevertheless, PmDAM1, PmDAM5, and PmDAM6 could form homo- and heteromeric complexes and act during the different stages of the dormancy cycle. These studies illustrate the composition of SVP-like and DAM complexes that have particular functions in flower development (Zhao et al., 2018a). Therefore, clarifying the nature of DAM and SVP-like protein complexes may help to better understand the integral function and potential regulation in flower bud differentiation and dormancy.

As a summary of the discussion above, it is interesting to find out the specific relationship between SVPs and DAMs and the neofunctionalization in this subclade. In this study, two *PmSVPs* and six *PmDAMs* originated from phylogenetic relationships were further analyzed. The expression patterns of eight genes in flower bud differentiation and dormancy were investigated by qRT-PCR. Furthermore, yeast two-hybrid assays were performed to examine the protein–protein interactions of *PmSVPs* and *PmDAMs*. Based on these results, we discussed the individual and combined function of *PmSVPs* and *PmDAMs* during flower bud development. A new model for flower organogenesis and dormancy was generated. This study may provide a theoretical basis for the improvement of flower phenotype and the regulation of flowering time in *P. mume*.

## Materials and methods

### Plant material

**Figure 1:**
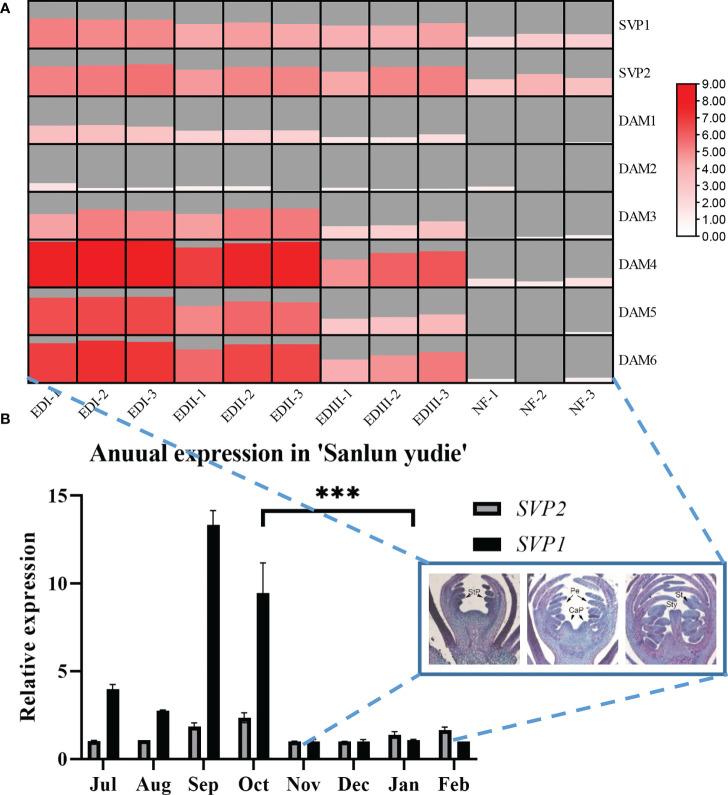
**(A)** Transcriptome data of *PmSVPs* and *PmDAMs* during four dormancy stages from 6 November 2015 to 8 March 2016. In the heat map, the samples considered were listed as follows: November 22 as endodormancy I (EDI), December 14 as endodormancy II (EDII), January 6 as endodormancy III (EDIII), and February 18 as natural flowering (NF). Data were scaled by log_2_, and the gray area displayed the scale size for each sample. **(B)** Annual expression patterns of *PmSVPs* in ‘Sanlun Yudie’ throughout floral growth and dormancy. The asterisks in the figure mean significance between samples.

**Figure 2:**
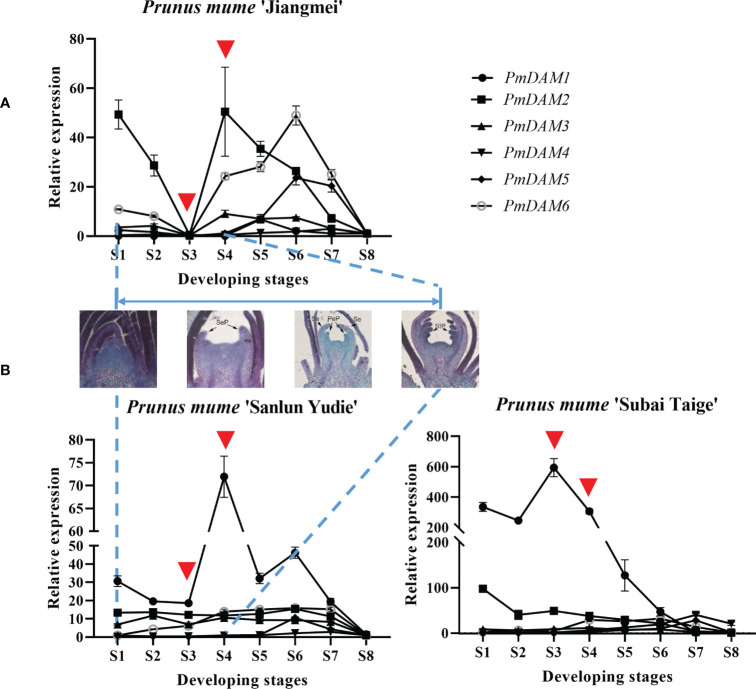
**(A, B)** Expression patterns of *PmDAMs* during the flower development of *P. mume* in the three cultivars. The middle figures show the section of S1–S4 stages. The red triangles located the core expression difference in the first four stages.

Mei cultivars with different flower types—’Jiangmei’, ‘Sanlun Yudie’, and ‘Subai Taige’—from Jiufeng International Plum Blossom Garden, Beijing, China (40°07′ N, 116°11′ E), were used in this study from July 2015 to February 2016. To analyze the expression pattern of *PmSVPs* and *PmDAMs* during flower bud differentiation and dormancy in three cultivars, a sample of each stage was collected every 30 days from July to February. Two samples at eight stages of early floral development with consistent appearance were collected every 57 days: one was immediately frozen in liquid nitrogen for RNA extraction, and the other was preserved in formaldehyde/acetic acid for the performance of paraffin sectioning (displayed in **Figures 1**, **2** and **Supplementary Figure S1**). Flower samples of ‘Sanlun Yudie’ planted in the Fujian Normal University were collected to clone *PmSVPs* and *PmDAMs*.

### Total RNA extraction and cloning of *PmSVPs* and *PmDAMs* sequences

Two *PmSVPs* and six *PmDAMs* have been identified in previous studies (Li et al., 2017; Zhao et al., 2018a). The total RNA was extracted using EASYspin plus plant RNA rapid extraction kit (RN38 Aidlab, Beijing), following the manufacturer’s directions. Potentially contaminating gDNA was removed using RNase-free DNase I (M6106, Aidlab, Promega). The cDNA was synthesized and purified using Fast Quant RT Kit (KR106, Tiangen, Beijing). Plasmids were extracted by normal methods. The primers and annealing temperature values of PCRs are presented in **Supplementary Table S1**.

### Genome acquisition and cis-acting element enrichment

The DNA sequences were extracted from *Prunus mume* Genome Project (http://prunusmumegenome.bjfu.edu.cn/). The SVP1 (Pm002166) and SVP2 (Pm022002) promoters were obtained from the position of 2,000 bp before the promoter initiation codon. Cis-acting elements were predicted by Promoter 2.0 Prediction Server (https://services.healthtech.dtu.dk/service.php?Promoter-2.0). The details are listed in **Supplementary Data 4**.

### Phylogenetic analyses

Two PmSVP proteins and six PmDAM proteins were aligned with 28 homologous protein sequences from other plants (eight in *P. persica*, five in *P. pseudocerasus*, seven in *Pyrus pyrifolia*, four in *M. domestica*, and four in *Camellia sinensis*) through DNAMAN 7.0 software with default parameters. The GenBank accession numbers of all sequences are shown in **Supplementary Data 2**. A phylogenetic tree based on the abovementioned proteins and the other 19 type II MADS-box proteins in *P. mume* (**Supplementary Data 3**) was constructed by using the maximum-likelihood method of MEGA11 program. The parameters of this tree were set to default, except for the bootstrap values which were set to 1,000.

### Quantitative real-time PCR and transcriptome data

The expression patterns of *PmSVPs* and *PmDAMs* in different flower bud development stages were determined, all by quantitative real-time PCR following previous methods. Primers were designed for cross-introns and ensured gene specificity. The primer amplification efficiency (standard curve) was calculated and guaranteed to be within 95%–105%. The intrinsic *PmPP2A* gene was used as the internal control, and the relative expression levels were computed using the 2^-ΔΔCt^ method (Ding et al., 2020). All qRT-PCR experiments were performed with three biological duplications, and each duplication was repeated in triplicate. The primers of qRT-PCR are shown in **Supplementary Table S2**. A comparison between samples and the drawings of relative expressions was conducted by GraphPad Prism; ANOVA was chosen as the statistical approach. The transcriptome data used for the expression were obtained from the dormancy research of *P. mume* (Zhang et al., 2018). The heat map was drawn by the software of TBtools (Chen et al., 2020).

### Yeast two-hybrid assays

The cDNA of *PmDAMs* and *PmSVPs* was amplified by PCR with gene-specific primers (**Supplementary Table S3**). These sequences were cloned into the pGBKT7 (bait) vectors and pGADT7 (prey) vectors (Clonetech, United States) at the EcoRI and BamHI sites, respectively, using InFusion HD Cloning Kit System. All baits were tested for autoactivation and toxicity. The yeast two-hybrid assays were performed according to the previous method (Zhao et al., 2018a). The screening for protein–protein interaction was applied in triplicate.

## Results

### PmSVPs displayed nucleotide compositional differences and extra phylogenetic cluster compared with PmDAMs

**Figure 3:**
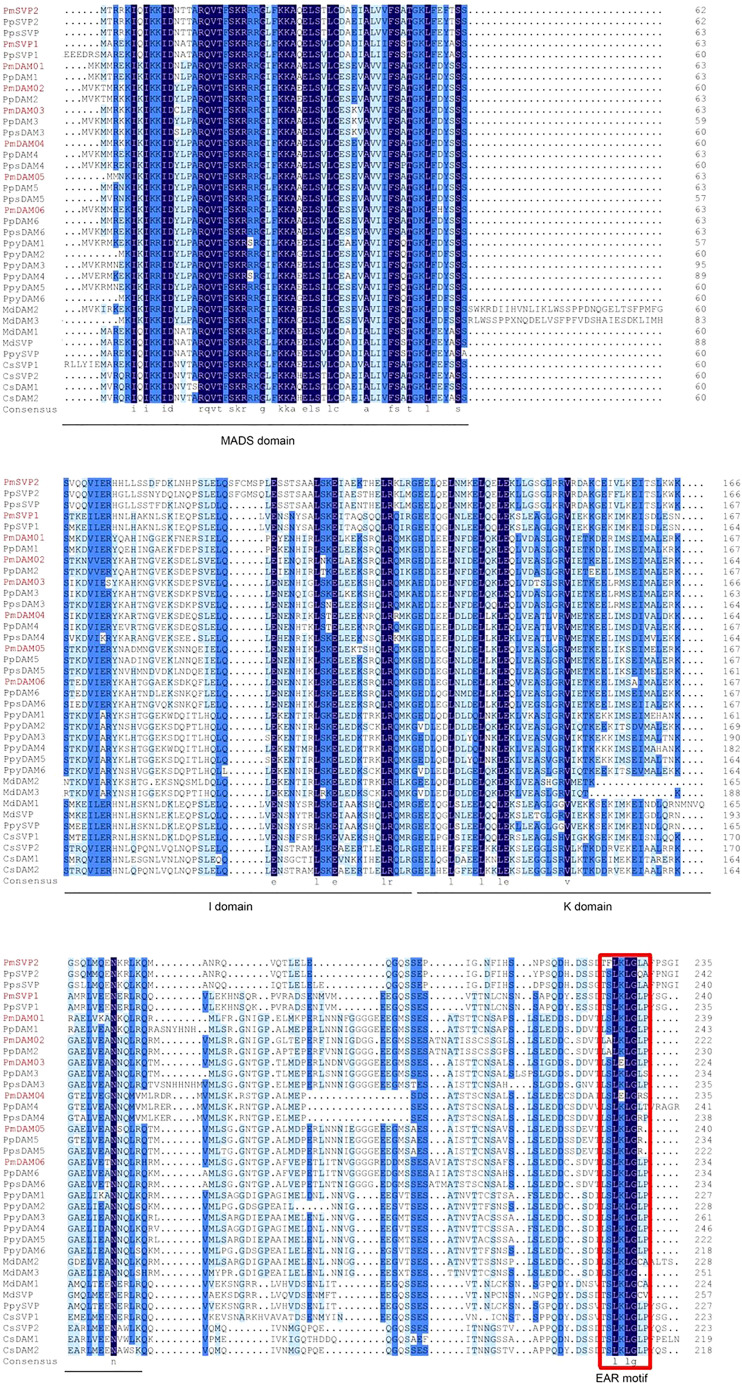
Multiple sequences alignment of *DAM* and *SVP* genes from *P. mume* and other species. The MADS domain, I domain, and K domain are shown by lines at the bottom of the alignment. The ethylene-responsive element-binding factor-associated amphiphilic repression motif is denoted by the red rectangle. The GenBank accession numbers of the genes used in the alignment are shown in **Supplementary Data S2**.

We obtained two *SVP* (*PmSVP1* and *PmSVP2*) and six *DAM* genes (*PmDAM1*, *PmDAM2*, *PmDAM3*, *PmDAM4*, *PmDAM5*, and *PmDAM6*) in the *P. mume* genome to ascertain their phylogenetic positions and sequence differences. *PmSVP1–2* had 687- and 672-bp open reading frames (ORFs), respectively, whereas *PmDAM1-6* contained 708-, 723-, 708-, 669-, 705-, and 726-bp ORFs. The coding sequences of eight genes were nearly similar, encoding between 222 and 241 amino acids (**Supplementary Data 1**). Multiple sequence alignment of SVPs and DAMs in different species was accomplished by DNAMAN program. The result showed that two PmSVPs and six PmDAMs proteins presented four major domains (**Figure 3**). MADS domain was highly conserved at N terminal, whereas K domain was moderately conserved and I domain was less conserved. In addition, eight protein sequences have conservative ERF-associated amphiphilic repression motifs at C terminal. Upon comparison with other homologous proteins, PmSVPs and PmDAMs have a higher homology with the SVPs and DAMs protein sequence of *P. percica* and *P. pseudocerasus* in the Rosaceae.

**Figure 4:**
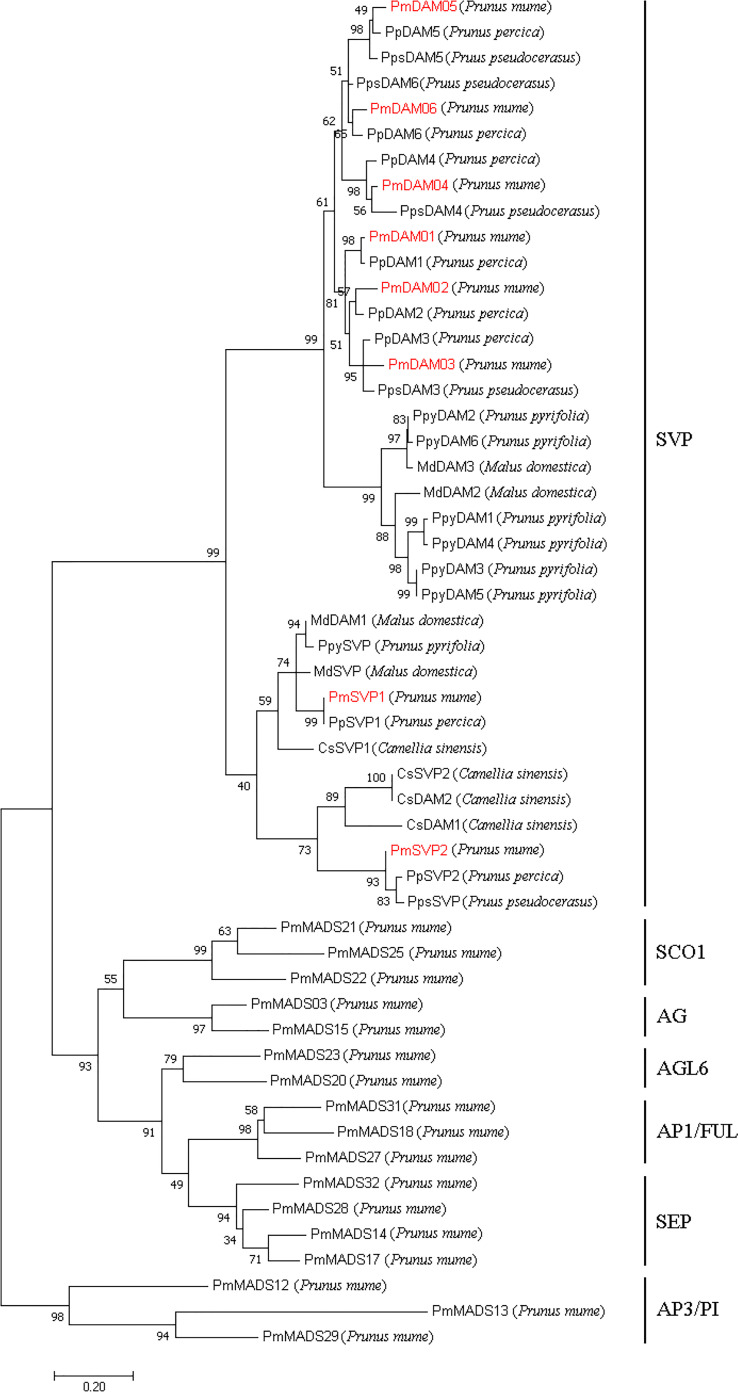
Phylogenetic tree of DAM and SVP proteins and other 17 type II MADS-box proteins in *P. mume*. The sequences of these proteins are shown in **Supplementary Data S3**. The types of *P. mume* MADS-box proteins in different clusters were named according to the phylogenetic analysis of the MADS-box gene family (Xu et al., 2014). The numbers above the branches represent the bootstrap value. DAMs and SVPs in *P. mume* are colored red.

The phylogenetic tree exposed that two *PmSVP*s and six *PmDAM*s belong to the SVP subclade of MADs-box gene family, and they were subdivided into two groups (**Figure 4**). On one hand, DAM group located in the proximal end of the evolutionary tree. On the other side, in the SVP group, PmSVP1 and PmSVP2 belong to two different branches, clustered with SVP and DAM from other species, respectively. In the far-side group, DAM proteins also gathered and subdivided into two groups. *PmDAM4*, *PmDAM5*, and *PmDAM6* formed a cluster, while *PmDAM1*, *PmDAM2*, and *PmDAM3* were congregated in the other, which has been discussed in the previous research (Zhao et al., 2018a). Consistently with the results of multiple sequence alignment, PmSVPs and PmDAMs were clustered with others from *P. persica* and *P. pseudocerasus*.

### The expression mode of *PmSVPs* and *PmDAMs* showed rhythmic regulation in floral differentiation rather than dormancy stages

According to the dormancy cycle of *P. mume*, dormancy occurs from September to October, and then the plant keeps dormant from November to January, finally breaking its dormancy in February. To explore the role of two *PmSVPs* and six *PmDAMs* in flower organ development and dormancy, the expression patterns of the eight genes were investigated by quantitative RT-PCR. From the annual relative expression pattern (**Figure 1B**), we found that *PmSVP1* displayed a rather high rhythm in September and October, and *PmSVP2* was expressed higher in the months of July to October. These two genes were active in warm months, and from November to February the expressions were quite low. Combined with the transcriptome data (**Figure 1A**), in the dormancy stage (EDI–EDIII), DAM gene, especially *PmDAM4-6*, reflected higher expressions than *PmSVP1–2.* This suggested that the main working members in the dormancy stage were DAM gene, and we also found that, in this period, the floral bud did little differentiation process (**Figure 1B**), the section pictures included basal tissues, and most processes finished in the months of July to October.

**Figure 5:**
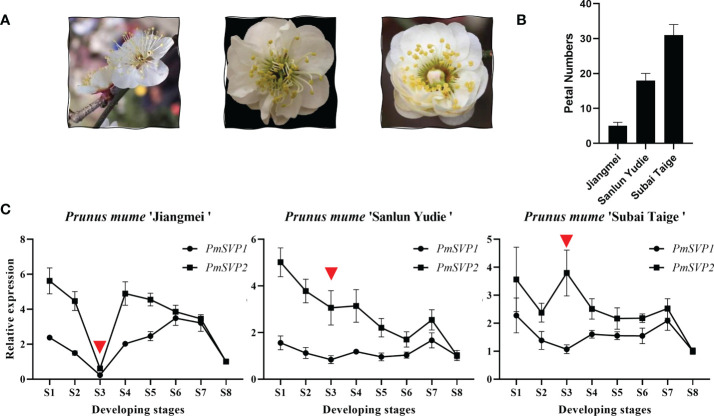
**(A)** Final shape of the three cultivars. **(B)** Petal numbers of the three cultivars. **(C)** Expression patterns of *PmSVPs* during the flower development of *P. mume*. Eight development stages of *P. mume* flower buds: undifferentiation (S1), flower primordium formation (S2), sepal initiation (S3), petal initiation (S4), stamen initiation (S5), pistil initiation (S6), ovule development (S7), and anther development (S8).

With the expectation into the development stages, paraffin sections were done to exhibit general flower bud differentiation (**Supplementary Figure S1**), we obtained eight typical development stages, in which the process of flower bud differentiation lasted from July to November. These included undifferentiated stage (S1), flower primordium differentiation stage (S2), sepal differentiation stage (S3), petal differentiation stage (S4), stamen differentiation stage (S5), pistil differentiation stage (S6), ovary development (S7), and pollen formation (S8). To show the function differences, we quantified eight *SVP* homologous genes in three cultivars, namely, ‘Jiangmei’, ‘Sanlun Yudie’, and ‘Subai Taige’. These three cultivars own similar characteristics (**Figure 5A**), except for the petal numbers. Indeed ‘Jiangmei’ has the least petals and ‘Subai Taige’ the most (**Figure 5B**). This last characteristic was the result of prime flower bud differentiation. *PmSVP1–2* were relatively highly expressed in the first seven stages of ‘Sanlun Yudie’ and ‘Subai Taige’ (**Figure 5C**), but as for ‘Jiangmei’, especially in S3 stage, the expression of *PmSVP1–2* was quite low. The said results showed accordance with the petal numbers of the three cultivars.

This phenomenon also happened to the expression of DAM genes. The expression of *PmDAM4–6* in different flower types was relatively low compared with *PmDAM1*. *PmDAM4*, *PmDAM5*, and *PmDAM6* showed similar expression profiles from stage 4 to stage 8 (S4–S8). The transcription of *PmDAM4* and *PmDAM5* was highly expressed in S5-S7, and *PmDAM6* had a high expression during S4–S7 (**Figure 2B**). *PmDAM1* performed noticeably well in the stages of S1–S8, and a clinal diversity was observed in S3 and S4 (**Figures 2A, B**), which is the core stage of petal formation (**Figure 2B**; S1–S4 paraffin sections). However, in ‘Jiangmei’, *PmDAM1* was less expressed with other DAMs in S3 stages. In the garret-like flower type ‘Subai Taige’, *PmDAM1–3* were relatively highly expressed in S1–S6, and *PmDAM1* reached a peak in S3. It is obvious that the expression levels of *PmDAM1* were significantly higher than those of the other genes and had similar patterns with ‘Sanlun Yudie’.

**Figure 6:**
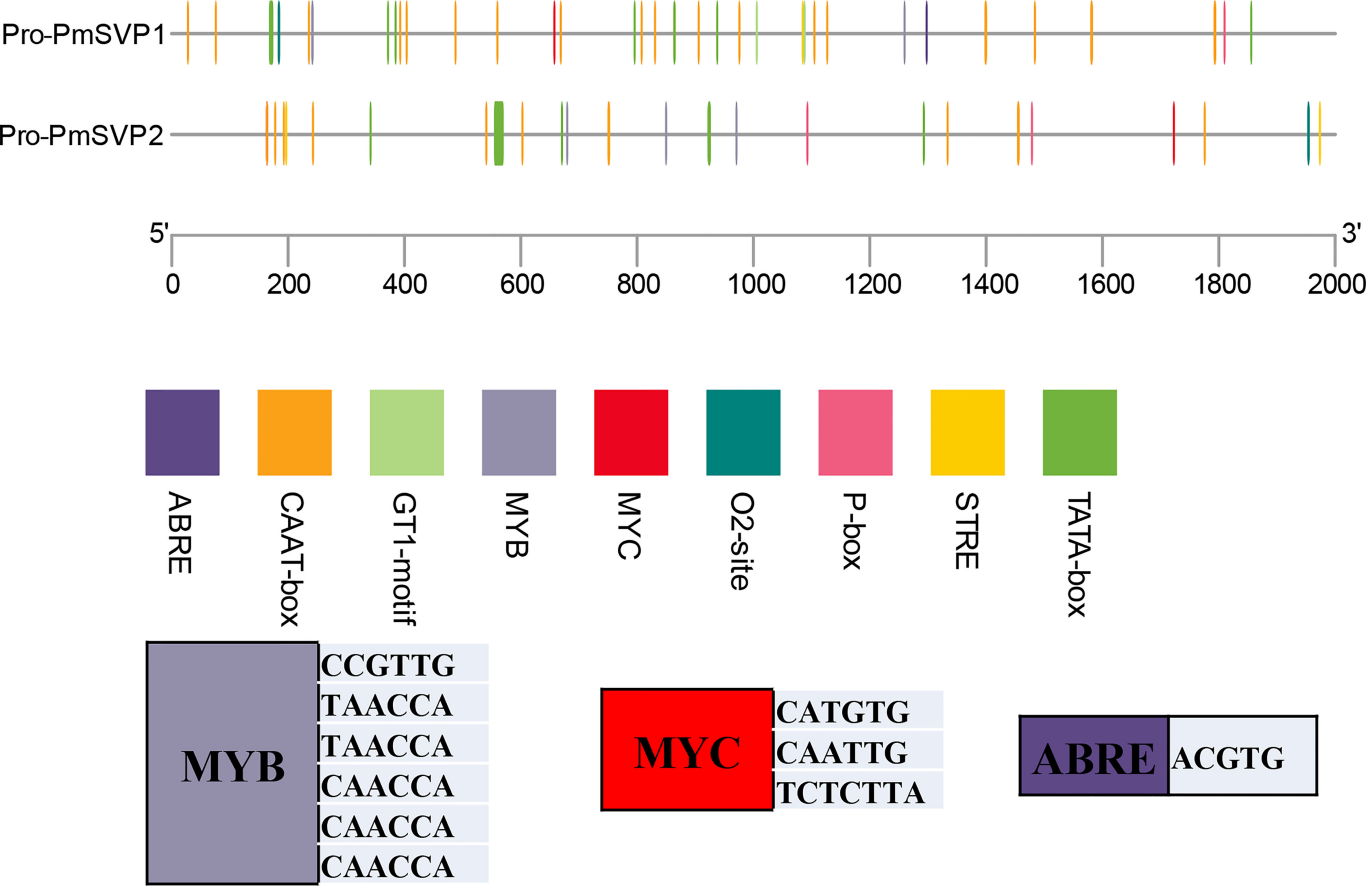
The 2k promoter elements of *PmSVPs.* Stubs listed from the 5′ end to the 3′ end show the location of each motif as recognized by the Plant Care online program. Colored blocks show the cis elements of different genes. At the bottom is the list of main recognition sequences.

The expression difference between SVPs may be the result of promoter elements. We obtained 2,000 nucleotide sequences and analyzed the binding elements (**Figure 6**). Some sites like MYB, MYC, and ABRE were found, and a total of 89 elements were displayed as short lines. In the elements, most were TAT-box, six were MYB binding sites, and two were MYC elements. One ABRE recognizing site was found in the promoter of PmSVP1 at the location of 1,296–1,301. This may be the reason that *PmSVP1* was expressed higher than *PmSVP2* in this research.

### Protein–protein interaction potency among SVP and DAM genes in *P. mume*

**Figure 7:**
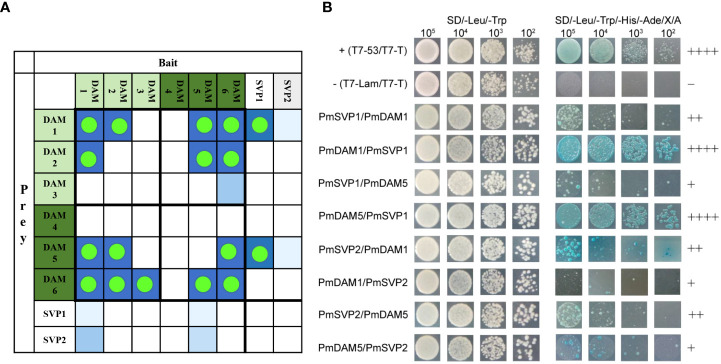
**(A)** The interaction summary among the DAM and SVP proteins, green dots highlighted the PPI results, blue blocks displayed the probability of the interactions. **(B)** The detailed yeast two-hybrid assays among PmSVPs and PmDAMs. To determine the interaction model of PmSVPs and PmDAMs, Yeast two-hybrid assays were performed. T7-53/T7-T was positive control, and T7-Lam/T7-T was negative control. The symbol (+) represents the capacity of the reaction. The more numbers of the symbol (+), stronger is the capacity of the reaction.

Yeast two-hybrid assays were performed to investigate the interaction relationships between proteins among PmSVPs and PmDAMs. All of the eight bait proteins had no autoactivation activity and toxicity from the results of growth in two-type auxotrophic medium, and the transformed yeast formed a bacterial colony with all four levels of initial concentration. PmSVP1, PmSVP2, PmDAM1, and PmDAM5 could form heterodimers with each other, including strong interactions and weak results (**Figure 7**). These heterodimers showed unequal interactive capability. PmDAM1 and PmDAM5 could strongly dimerize with PmSVP1. PmSVP1 interacted with PmDAM1 moderately, while the interaction between PmSVP1 and PmDAM5 was weak. The abilities of PmSVP2 to interact with PmDAM1 or PmDAM5 were moderate. The Y2H confirmed the dimerization between each two molecules.

## Discussion

### Alienation in expression differentiated the function of *PmDAMs* and *PmSVPs*

*SVP*-like genes, members of MADS-box gene, evolved into multiple paralogs in a wide range of species. In *P. mume*, there are two *SVP* genes (*PmSVP1* and *PmSVP2*) and six tandemly duplicated *DAM* genes (*PmDAM1*–*PmDAM6*) found in the genome data. These eight genes are conserved with other *SVP* genes in Rosaceae, especially showing quite similar structures with *SVP*-like genes in *P. percica*. They comprised a characteristic arrangement of four domains. More differences were observed for the C-terminal region, which assign these proteins with binding capacity of different intensity (Smaczniak et al., 2012). Members from *P. mume*, *P. percica*, and *P. pseudocerasus* were congregated; they are reported to undergo their own duplication events in *Prunus*, which are different from the whole-genome duplication events shared by *Malus* and *Pyrus* (Jimenez et al., 2009). Furthermore, *SVP* genes and *DAM* genes were separated into two groups. The segregation of *PmDAMs* and *PmSVPs* into different clusters indicated that neofunctionalization and subfunctionalization may occur between these genes during the gene duplication events in *Prunus*. Similar results were presented in peach. The tandem duplication of *PmSVPs* and its ability to form a protein complex lead to functional diversity during evolution, which may have been beneficial for responding to variable environments to regulate flowering. The SVP-like gene family has been shown to perform diverse functions between different species. In *Arabidopsis*, *SVP* and *AGL24* are implicated in floral transition and development. *SVP* genes suppress the flowering process (Yu et al., 2004). Overexpression of *PmSVP1* and *PmSVP2* in *Arabidopsis* resulted in floral abnormalities, suggesting similar roles in floral development in *P. mume* (Li et al., 2017). The *PmSVP* and *PmDAM* genes have also been implicated in dormancy regulation. The expression profile could help us to further infer their function during flower development and dormancy. From July to November, two Mei cultivars *PmSVP1–2* were relatively highly expressed in the first seven stages, indicating that *PmSVP1–2* were likely involved in the whole process of flower bud differentiation. As we have reported in the previous research (Zhao et al., 2018a), the expression levels of *PmDAM1–3* were relatively high in the flower bud differentiation process, while *PmDAM4–6* have a relatively high expression in the overlapping stage when the flower bud went through differentiation and dormancy process in ‘Subai Taige’. These seasonal expression profiles had a high degree of similarity to those in ‘Sanlun Yudie’ and other plants such as peach and apple. In the early flower bud differentiation of proliferation flower ‘Subai Taige’ and triple-petal flower ‘Sanlun Yudie’, *PmDAM1* was significantly detected in S3 and S4, respectively. This suggested that *PmDAM1* may be involved in sepal differentiation and petal initiation. As the temperature drops, *PmDAM4–6* gradually increased. *PmDAM5* was high in S7 in ‘Subai Taige’ and S6 in ‘Sanlun Yudie’. This gene may regulate pistil variation. *PmDAM1* and *PmDAM5* might be the main reasons for the formation of multiple petals and upper flower. Meanwhile, *PmSVP1–2* and *PmDAM4–6* were prominently expressed in late flower bud differentiation, the overlapping flower bud differentiation, and the dormancy process.

### Interaction evolved from closely related genes accelerated neofunctionalization in opposite functions

**Figure 8:**
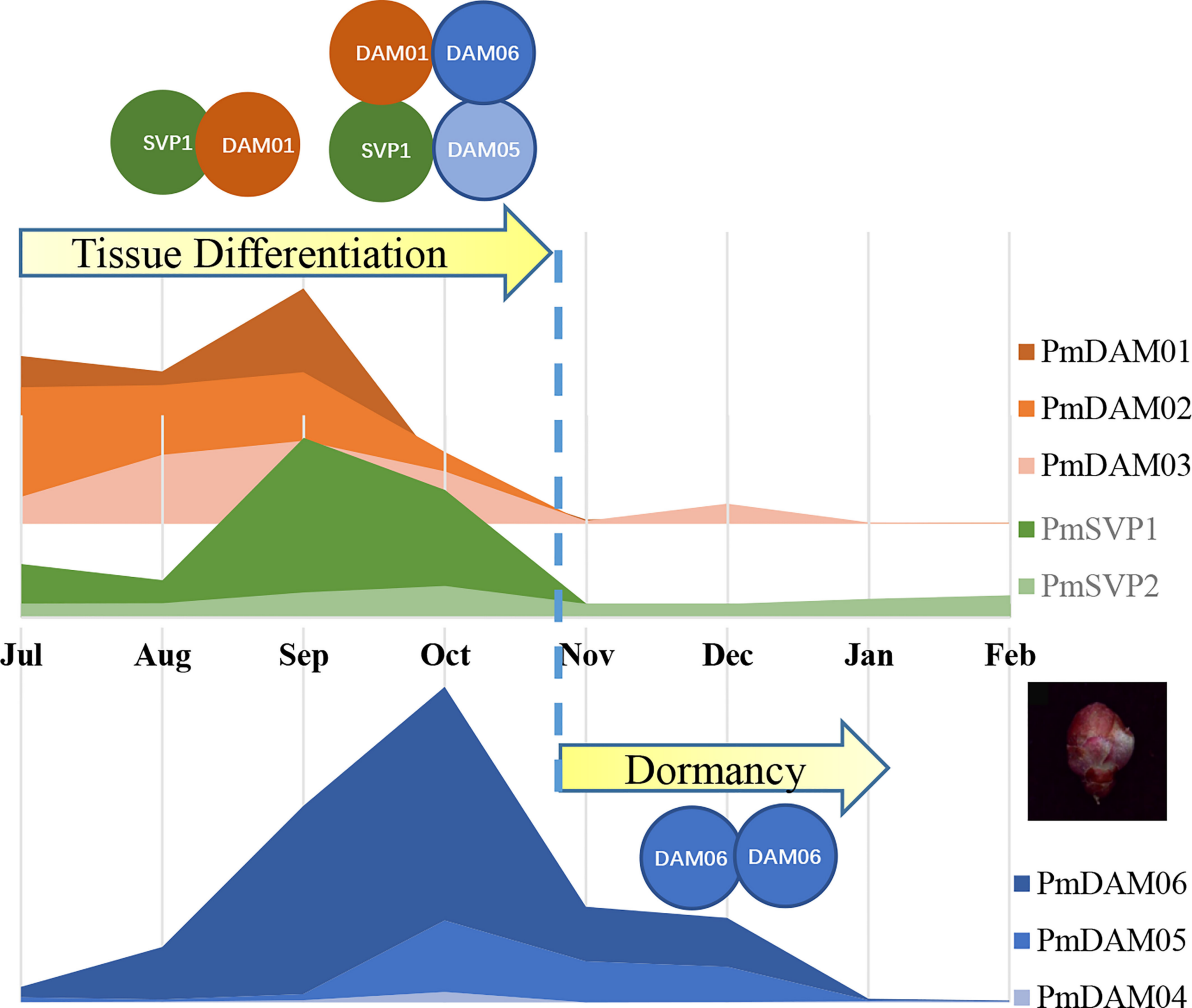
The assumed regulatory model for PmSVPs and PmDAMs in floral tissue differentiation and dormancy. Orange and blue lumps represent the expression changes of DAM 1–6, while green lumps stand for the expression of SVP 1–2. The curves were drawn based on the mean value of relative expression. The collision between circles displays the protein–protein interaction among SVPs and DAMs.

PPI were considered extremely conserved in basal eudicots, even for the most recent common ancestor of extant angiosperms (Liu et al., 2010; Li et al., 2015). While containing an interaction motif, variation in MADS-box may be a potent driver of floral developmental evolution through protein–protein interactions (Bartlett, 2017). PmSVPs and PmDAMs could form heterodimers with each other in *P. mume* by yeast two-hybrid assay; a similar result was also recently demonstrated in wheat. TFs could act in protein complexes, and both their activity and the sites to which they bind are likely to be strongly modulated by the presence or absence of other members of the complex (Mateos et al., 2015). Here we captured the functions of PmSVPs and PmDAMs during flower organogenesis and dormancy, especially for PmSVP1 and PmDAM1, combining this research and previous studies. In *P. mume*, flower bud differentiation begins in July, and PmDAM1 forms a homologous dimer with PmSVP2. During the next stage, PmDAM2 began to accumulate, PmDAM1 may be involved in the flower primordial and sepal petal differentiation by forming a homologous dimer or heterodimer with PmDAM2 and PmSVP2. Then, PmDAM3 and PmDAM6 started to participate in petal and stamen differentiation. PmDAM3 could form dimers with PmDAM6 and PmSVP2, which may mainly regulate the double petals. For pistil and ovary development, also the overlapping of flower bud differentiation and dormancy, PmDAM5 and PmDAM6 act in these stages as the temperature drops. PmDAM5, PmDAM6, and PmSVP1–2 could form a heterodimer to transform plants from flower bud differentiation to dormancy. Lastly, a homodimer of PmDAM6 was in the lead during the dormancy stage. Taken together, with the change of temperature, a dynamic interaction between PmSVPs and PmDAMs could regulate floral development and then switch into dormancy. However, a vexatious problem comes up—the aggregation morphology—whether the SVPs and DAMs functioned as dimers or multimers was still not verified in our experiment. Previous research supplied hints that SVP could form a higher complex due to the flexible C-terminal residues (Wang et al., 2015). This may seriously affect the detailed regulation manners, and in the further research, interactome or Y3H may help deepen our insights into this complex action. Therefore, we raised a hypothesis based on our foundation to describe the roles that PmSVPs and PmDAMs lead to promote tissue differentiation and plant dormancy (**Figure 8**). After the floral bud appearance, SVP1 gradually accumulated and mingled in the complex by DAM1 and 2 by grabbing DAM1. This led the tissue differentiation to proceed. Between September and October, with the temperature falling, DAM genes began to induce. The presence of SVP1 made it possible to maintain a higher complex, including SVP1, DAM1, DAM5, and DAM6. This helped not to perform the full function of DAM6 and DAM5, but we believed that the protein commixture also slowed down the cell activities. When the temperature finally came to a certain degree, the expressions of SVP were stopped. The disappearance of SVP released the complex. Thus, the domination of DAM6 facilitated the termination of physical activity and the inner situation switch, including dehydration and glucose accumulation, which help plant cells survive in a freezing environment.

## Conclusions

After the investigation of the evolutionary expression profiles and combinatorial activity of PmSVPs and PmDAMs, we rebuild the interaction model, taking a glimpse on their crucial roles in flower development control. Their activities change when they act individually or as a complex. This behavior confers flexibility to the regulatory network to prevent the premature development and growth of flowers during the unfavorable winter periods. This study could provide additional understanding on the comprehensive functions of PmSVPs and PmDAMs in the switch of flower bud development and dormancy, especially illustrating that PmSVP1 promoted floral tissue differentiation by maintaining the complex with PmDAM1. This research could provide a reference for revealing how floral morphological diversity arose and how dormancy regulation was effected.

## Data availability statement

The datasets presented in this study can be found in online repositories. The names of the repository/repositories and accession number(s) can be found in the article/**Supplementary Material**.

## Author contributions

KZ and YuZ designed the whole experiments; KZ, YaZ, YuZ wrote the manuscript. YaZ, MH, YT, RZ, MS collected necessary samples; YuZ, XL, YZ, RZ, MS, handle the experiments and analyzed the data. KZ performed the analyses of sequencing data and was responsible for figure compiling and organization. All authors read and approved the final manuscript.
