# Supplementary material for: The collaborative mode by PmSVPs and PmDAMs reveals neofunctionalization in the switch of the flower bud development and dormancy for Prunus mume
Source: Front Plant Sci. 2022 Dec 6;13:1023628. doi: 10.3389/fpls.2022.1023628 (PMC9763448; doi:10.3389/fpls.2022.1023628)
Supplement: Supplementary file 1 [file DataSheet_1.docx]

Supplementary Material

**The collaborative mode by** ***P******mSVPs* and *PmDAMs* reveals neofunctionalization in the switch of the flower bud development and dormancy for *Prunus mume***

Zhao Kai^1*^ Yuzhen Zhou^2^ Zheng Yan^2^ Zheng Rui-yue^2^ Meijuan Hu^2^ Yan Tong^2^ Xianmei Luo^1^ Yangting Zhang^2^ Ming-li Shen^1^

1 College of Life Sciences, Fujian Normal University, Fuzhou, 350117

2 College of Landscape Architecture, Ornamental Plant Germplasm Resources Innovation & Engineering Application Research Center at College of Landscape Architecture, Key Laboratory of National Forestry and Grassland Administration for Orchid Conservation and Utilization at College of Landscape Architecture, Fujian Agriculture and Forestry University, Fuzhou, China, 35002

*** Correspondence: zhaokai@fjnu.edu.cn**

# 1 Supplementary Figures and Tables

## 1.1 Supplementary Figures

**Supplementary Figure 1.** General flower bud differentiation sections of *P. mume*. The flower bud development was divided into eight stages (S1-8): undifferentiation (S1), flower primordium formation (S2), sepal initiation (S3), petal initiation (S4), stamen initiation (S5), pistil initiation (S6), ovule development (S7), anther development (S8). The letters had different meanings. FP: Flower primordiu; SeP: Sepal primordium; Se: Sepal; PeP: Petal primordium; Pe: Petal; StP: Stamen primordium; St: Stamen; CaP: Carpel primordium; Ca: Carpel; Sty: Style; An: Anther; F: Filament; Ova: Ovary; Ovu: Ovule; Po: Pollen.


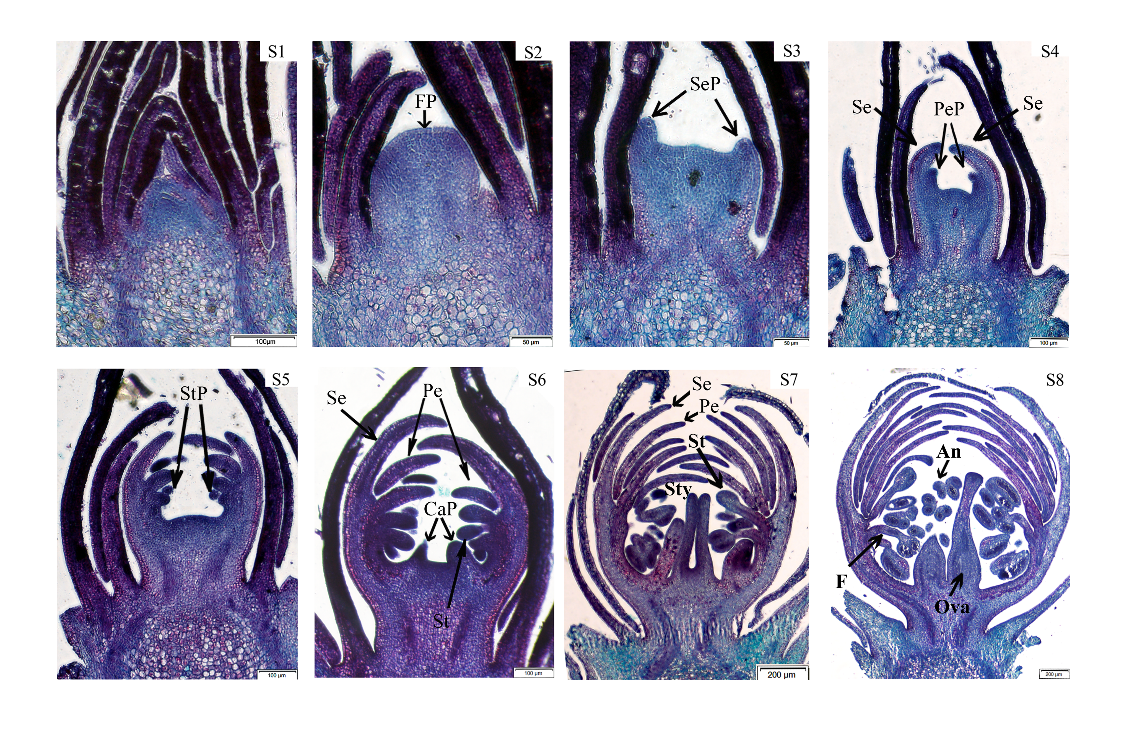


## 1.2 Supplementary Tables

**Supplementary Table 1.**Primers used for cloning.

| **Gene** | **Forward primer** | **Reverse primer** | **Annealing temperature** |
| --- | --- | --- | --- |
| PmSVP1 | 5'AGATCTATGGCGAGGGAGAAGATTCAGA3' | 5'CACGTGTTAACCAGAGTAAGGTAACCCCAAT3' | 61 ℃ |
| PmSVP2 | 5'AGATCTATGACGAGGAGGAAAATCCAGATCAA3' | 5'GGTGACCTCATATCCCGCTAGGAAAAGCT3' | 60 ℃ |
| *PmDAM1* | 5'ATGAAAATGATGAGGGAGAAG3' | 5' TTATGGAAGCCCCAGTTTGAG3' | 52 ℃ |
| *PmDAM2* | 5'ATGGTGAAGACGATGAGGAAG3' | 5' TTAGGGAAGCCCCAGTTTGAG3' | 56 ℃ |
| *PmDAM3* | 5'ATGATGAGGAAGAAGATCAAG3' | 5' TTAAGGAAGCCCCAGTTCGAG3' | 51 ℃ |
| *PmDAM4* | 5'ATGGTGAAAATGATGAGGGAG3' | 5' TTAGGAACGCCCCAGTTTGAG3' | 54 ℃ |
| *PmDAM5* | 5'ATGATGAATAAGATCAAGATC3' | 5' TTAACGCCCCAGTTTGAGAGA3' | 47 ℃ |
| *PmDAM6* | 5'ATGGTGAAAATGATGAGGGAG3' | 5' CTAGGGAAGCCCCAGTTTGAG3' | 59 ℃ |

## Supplementary Table 2. Primers used for real-time quantitative RT-PCR.

| **Gene** | **Forward primer** | **Reverse primer** |
| --- | --- | --- |
| *PP2A2* | ATATAGCTGCTCAGTTCAACC | AAAAACAGTCACCACATTCTT |
| *PmSVP1* | CCACTGGAAAACTCTTTGAATACG | CAAGAGATGGTTGTTCTATTTTCG |
| *PmSVP2* | TGTGATGCTGAGATTGCTCTTGTAG | CAGGTGATGCCTTTCAATTACTTG |
| *PmDAM1* | AGTATGAAGGATGTTATTCAA | CTTAAGTTCCTTGCTCAATCT |
| *PmDAM2* | AACCAGCTACGGCAGAGGATG | AGATTCAGATGACATGCCTT |
| *PmDAM3* | TCGGATTGAGCAAGGAACTGG | CATTCTCAGTTCTTCCTTTGT |
| *PmDAM4* | ACCCTTGTCCGTGTGATGGAA | ATCACCATCTGATTGTTGCCT |
| *PmDAM5* | AGGCTGAATAATAATATTGAA | TTAACGCCCCAGTTTGAGAGA |
| *PmDAM6* | AACCAACAACCAGTTAAGGCATA | CAATTACGGCAGATTCAGATGA |

**Supplementary Table 3.** Primer used in PCR reaction for Y2H assays.

| **Gene** | **Sequence 5’-3’** |
| --- | --- |
| BK-PmSVP1-F | CATGGAGGCCGAATTCATGGCGAGGGAGAAGATTCA |
| BK-PmSVP1-R | GCAGGTCGACGGATCCTTAACCAGAGTAAGGTAAC |
| BK-PmSVP2-F | CATGGAGGCCGAATTCATGACGAGGAGGAAAATCCA |
| BK-PmSVP2-R | GCAGGTCGACGGATCCTCATATCCCGCTAGGAAAAG |
| BK-PmDAM1-F | CATGGAGGCCGAATTCATGAAAATGATGAGGGAGAAG |
| BK-PmDAM1-R | GCAGGTCGACGGATCCTTATGGAAGCCCCAGTTTGAG |
| BK-PmDAM5-F | CATGGAGGCCGAATTCATGATGAATAAGATCAAGATC |
| BK-PmDAM5-R | GCAGGTCGACGGATCCTTAACGCCCCAGTTTGAGAGA |
| AD-PmSVP1-F | GGAGGCCAGTGAATTCATGGCGAGGGAGAAGATTCA |
| AD-PmSVP1-R | CGAGCTCGATGGATCCTTAACCAGAGTAAGGTAAC |
| AD-PmSVP2-F | GGAGGCCAGTGAATTCATGACGAGGAGGAAAATCCA |
| AD-PmSVP2-R | CGAGCTCGATGGATCCTCATATCCCGCTAGGAAAAG |
| AD-PmDAM1-F | GGAGGCCAGTGAATTCATGAAAATGATGAGGGAGAAG |
| AD-PmDAM1-R | CGAGCTCGATGGATCCTTATGGAAGCCCCAGTTTGAG |
| AD-PmDAM5-F | GGAGGCCAGTGAATTCATGATGAATAAGATCAAGATC |
| AD-PmDAM5-R | CGAGCTCGATGGATCCTTAACGCCCCAGTTTGAGAGA |

# 2 Supplementary Data

**Supplementary Data1.** The CDS sequences of two *PmSVPs* and six *PmDAMs*.

>PmSVP1

atggcgagggagaagattcagatcaagaagatcgacaacgccacggcgaggcaggtgaccttttccaagcggaggagagggcttttcaagaaggctcaggagctctccgttctctgtgatgcagatattgctcttatcatcttttcttccactggaaaactctttgaatacgccagctccagcacgaaggaaattctagaacgtcacaacttgcacgcaaagaatctctcgaaaatagaacaaccatctcttgagttacagctagtggagaacagcaactactctgcgttgagcaaggaaattacagcacaaagtcaacaacttaggcagataaggggagaagaaatccaaggattaaatttggaagaattgcagcaactggagaagtctcttgaagctggattgggccgcgtaatagagaaaaagggtgaaaagattatgaaagagatcagcgatctcgaaagcaatgcgatgcgattggttgaagagaatgaacggctgagacagcaagtgctggagaaacataatagccagaggccggttcgggccgattcagaaaacatggttatggaggagggtcagtcatcagagtctgtcaccaccaacctctgcaactctaacagcgctccgcaagactatgagagctcagatacatctctcaaattggggttaccttactctggttaa

>PmSVP2

atgacgaggaggaaaatccagatcaagaagatcgacaacacaacggcgaggcaggtgacgttttcgaaaaggaggagagggcttttcaagaaagcccaggagctctctactctctgtgatgctgagattgctcttgtagtcttctccgctactgggaagctctttgaattcaccagctccagcgtgcaacaagtaattgaaaggcatcacctgctttcttccgattttgacaagttgaatcatccatctcttgagctgcagtccttttgtatgtctccgcttgagagcagtacttccgccgcattgagcaaggaaattgcggagaaaacacatgagctgaggaagctaaggggagaagaactccaagaactaaacatgaaagagttgcaggaactagagaaactgctcggatcaggattgaggcgtgttagagatgcaaagtgtgaaattgttctgaaggagatcacctctcttaagtggaagggatcccaacttatgcaagaaaacaagcgattgaagcagatggcaaaccgacaggtccaaacacttgaacttgaacaaggccaatcctccgagccaataggcaatttcatccattcaaacccttcgcaagaccacgacagctctgacacttttctcaagttggggttagcttttcctagcgggatatga

>PmDAM1

atgaaaatgatgagggagaagatcaagatcaagaagattgacaacttgcctgcaaggcaagtgaccttctcaaagaggaggagagggatcttcaagaaagctgcagagttatctgttctgtgtgaatctgaggtggcagttgtcatcttttctgctactggcaagctttttgattattcaagctcaagtatgaaggatgttattgaaaggtaccaagcgcacataaatggtggtgaaaaatttaacgaacggtctattgagttgcagccagagtatgaaaaccacatcagattgagcaaggaacttaaggagaagagccgccagctgaggcagatgaaaggagaggatcttgaagagctgaattttgatgagttgcagaagttagaacaactggtggatgcaagccttggccgtgtgattgaaactaaggacgaacggattatgagtgagattatggcacttgaaagaaagagagctgagcttgtaaaagccaacaaacagctaaggcagaggatgttattcagaggaaatattggacctgagcttatgaagccggagaggttgaataataattttggtggtggaggagaagaagaaggtatgtcatctgaatctgctacctccaccacctgcaacagtgctccgagtctctctcttgaagatgactccgacgacgtcactttatctctcaaactggggcttccataa

>PmDAM2

atggtgaagacgatgaggaagaagatcaagatcaagaagattgactacttgcctgcaaggcaggtgaccttctcaaagaggaggagagggatcttcaaaaaagctgaggagctatctgttctgtgtgaatctgaggtggcagttgtcatcttttctgctactggcaagctttttgattattcaagctcaagtacaaagaatgttgttgaaaggtataaagcgcacacaaatggtgtcgaaaaatcggacgaactgtctgttgagctgcagctagaaattgaaaaccagatcagattgaacaaggaacttgcggagaagagccgccagctgaggcagatgagaggagaggatcttgaagagctgaatattgatgagttgcagaagttagaacaactggtggaggcaagccttggccgtgtgattgaaactaaggaagaactgattatgagtgagattatggcacttgaaagaaagggagctgagctggtagaagccaacaaccagctacggcagaggatggtgatgttatccagaggaaatattggacctgggcttacggagccggagaggttcattaataatattggagatggaggagaagaaggcatgtcatctgaatctgccacaaatgcaaccatcagcagctgcagcagtggtctcagtctctctcttgaagatgactgctcagacgtcactttagctctcaaactggggcttcccTaa

>PmDAM3

atgatgaggaagaagatcaagatcaagaagattgattgcttgcctgcaaggcaggtgactttctcaaagaggagaagagggatcttcaagaaagctgcagagctatctgttctgtgtgaatctaaggtggcagttgtcatattttctgctactggcaagctttttgattattcaagctcaagtatcaaggatgttattgaaagctacaaagcgcacaaaaatggtgtcaaaaaatcggacgaaccgtctgttgagctacagctagagaatgaaaatcacatcggattgagcaaggaactggaggagaagagccatcagctgaggcagatgaaagcagaggatcttgacgagctgaattttgatgagttgcagaagttagaacaactggtggacacaagccttagccgtgtgattgaaacaaaggaagaactgagaatgagtgagattatggcacttgaaagaaagggagctgagctggtagaagccaacaaccagctaaagcagacgatggtgatgttatccggaggaaatactggacctacgcttatggatccggagaggttgaatgataatgtcggaggtggaggagaagaagaaggcatgtcatctgaatctgctatctccaccacctgcaacagtgctctcagtctctccattggagatgactccgacgacgtcactttatctctcgaactggggcttccttaa

>PmDAM4

atgatgaggaagaagatcaagatcaagaagattgactacctgccagcaaggcaggtgacattctcaaagaggagaagagggatcttcaagaaagctgcagagctatctgttctgtgtgaatctgaggtggcagttgtcatcttttctgctactggcaagctttttgattattcaagctcaagtatcaaggatgttattgaaaggtacaaagcgcgcacaaatggtgtcgaaaaatcggatgaacagtctcttgagctgcagctggagaatgaaaaccgcatcaaactcagtacggaactcgaggagaagaaccgccagctgaggcggatgaaaggtgaggatcttgaagagctggatctggatgagttgctgaagttggaacaactggtggaagcaacccttgtccgtgtgatggaaactaaggaagaactgattatgagtgatattgtggcacttgataaaaagggaactgagctggtagaaggcaacaatcagatggtgatgttaagggacaggatggtgatgttatccaaaagaagtaccggacctgcgcttatggagccatctgactctgctacctccaccagctgcaacagtgctctgagtctttctcttgaagatgaatgctccgacgacgccattttatctctcgaactggggcgttcctaa

>PmDAM5

atgatgaataagatcaagatcaagaagattgactacttgcctgcaaggcaggtgaccttctcaaaaaggagaagagggctcttcaagaaagctgcagagctatctgttctgtgtgaatctgaggtggcagttgtcatcttttctgccactggcaagctttttgattattcaagctcaagtaccaaggatgttattgaaaggtacaacgcagacatgaatggtgtcgaaaaatcgaacaatcaagagattgagctgcagctggagaatgaaaaccacatcaaactgagtaaggaactcgagaagacgagccaccagctgaggcagatgaaaggtgaggatcttgaagggctgaatctggatgagttgctgaagttggaacaactggtggaagcaagccttggccgtgtcatggaaactaaggaagagctgattaagagtgagattatggaactcgaaagaaagggagctgagctagttgaagccaacagccagctaaggcagacgatggtgatgttatccggaggaaatactggacctgcgcttatggatccggagaggctgaataataatattgaaggtggaggagaagaagaaggcatgtcagctgaatctgctatctccaccacctgcaacagtgctgtcagtctctctcttgaagatgactcctccgatgaggtcactttgtctctcaaactggggcgttaa

>PmDAM6

Atggtgaaaatgatgagggagaagatcaagatcaagaagattgactacctgccagcaaggcaggttaccttttcaaagagaagaagagggctcttcaagaaagctgcagagctatcggttctgtgtgaatctgaggtggctgtcgtcatcttttctgccactgacaagctctttcattattcaagctcaagtaccgaggatgttattgaaaggtacaaagcgcacacaggtggtgccgaaaaatcagacaaacagtttcttgagctgcaactggagaatgaaaacaacatcaaactgagtaaggaactcgaggagaagagccgccagctgaggcagatgaaaggtgaggatcttgaagggctgaatctggatgagctgctgaagttagaacaagtggtggaagcaagccttggccgtgtgatagaaactaaggaagagctgattatgagtgcgattatggcactggagaaaaagggagctgagctggtagaaaccaacaaccagttaaggcataggatggtgatgttatccggaggaaatactggacctgcgtttgtggagccggagacgttgattactaatgttggaggtggaggacgagaagacgacatgtcatctgaatctgccgtaattgccacctccaccagctgcaacagtgctttcagtctctctcttgaagatgactgctccgatgtcactttatctctcaaactggggcttccctag

**Supplementary Data2.** The GeneBank accession numbers of genes used in alignment.

PmSVP1 (*P. mume*, AML81015.1); PmSVP2 (*P. mume*, AML81016.1); PpSVP1 (*P. persica*, XP_020422316); PpSVP2 (*P. persica*, XP_020409383); MdSVP (*M. domestica*, NP 001280915); PySVP (*P. pyrifolia*, AJW29050); PpsSVP (*P. pseudocerasus*, AKM27770); CsSVP1 (*C. sinensis*, XP_028064640.1); CsSVP2 (*C. sinensis*, XP_028076077.1); PmDAM1 (*P. mume*, BAK78921); PmDAM2 (*P. mume*, BAK78922); PmDAM3 (*P. mume*, BAK78923); PmDAM4 (*P. mume*, BAK78924); PmDAM5 (*P. mume*, BAK78920); PmDAM6 (*P. mume*, BAH22477); PpDAM1 (*P. persica*, ABJ96361); PpDAM2 (*P. persica*, ABJ96363); PpDAM3 (*P. persica*, ABJ96364); PpDAM4 (*P. persica*, ABJ96358); PpDAM5 (*P. persica*, ABJ96359); PpDAM6 (*P. persica*, ABJ96360); PpsDAM3 (*P. pseudocerasus*, AIU94275.1); PpsDAM4 (*P. pseudocerasus*, AIU94276.1); PpsDAM5 (*P. pseudocerasus*, AIU94277.1); PpsDAM6 (*P. pseudocerasu*s, AIU94278.1); PpyDAM1 (*P. pyrifolia*, BAM74184.1); PpyDAM2 (*P. pyrifolia*, BAM74183.1); PpyDAM3 (*P. pyrifolia*, BAM74167.1); PpyDAM4 (*P. pyrifolia*, BAM74166.1); PpyDAM5 (*P. pyrifolia*, BAI48075.1); PpyDAM6 (*P. pyrifolia*, BAI48074.1); MdDAM1 (*M. domestica*, AJW82923.1); MdDAM2 (*M. domestica*, AJW82922.1); MdDAM3 (*M. domestica*, AJW82921.1); CsDAM1 (*C. sinensis*, AIK35210.1); CsDAM2 (*C. sinensis*, AIK35209.1).

**Supplementary Data3.** The protein sequences of SVP and DAM proteins and other 17 type II MADS-box proteins in *P. mume*.

>PmSVP1

MAREKIQIKKIDNATARQVTFSKRRRGLFKKAQELSVLCDADIALIIFSSTGKLFEYASSSTKEILERHNLHAKNLSKIEQPSLELQLVENSNYSALSKEITAQSQQLRQIRGEEIQGLNLEELQQLEKSLEAGLGRVIEKKGEKIMKEISDLESNAMRLVEENERLRQQVLEKHNSQRPVRADSENMVMEEGQSSESVTTNLCNSNSAPQDYESSDTSLKLGLPYSG*

>PmSVP2

MTRRKIQIKKIDNTTARQVTFSKRRRGLFKKAQELSTLCDAEIALVVFSATGKLFEFTSS

SVQQVIERHHLLSSDFDKLNHPSLELQSFCMSPLESSTSAALSKEIAEKTHELRKLRGEE

LQELNMKELQELEKLLGSGLRRVRDAKCEIVLKEITSLKWKGSQLMQENKRLKQMANRQVQTLELEQGQSSEPIGNFIHSNPSQDHDSSDTFLKLGLAFPSGI*

>PmDAM1

MKMMREKIKIKKIDNLPARQVTFSKRRRGIFKKAAELSVLCESEVAVVIFSATGKLFDYS

SSSMKDVIERYQAHINGGEKFNERSIELQPEYENHIRLSKELKEKSRQLRQMKGEDLEEL

NFDELQKLEQLVDASLGRVIETKDERIMSEIMALERKRAELVKANKQLRQRMLFRGNIGP

ELMKPERLNNNFGGGGEEEGMSSESATSTTCNSAPSLSLEDDSDDVTLSLKLGLP*

>PmDAM2

MVKTMRKKIKIKKIDYLPARQVTFSKRRRGIFKKAEELSVLCESEVAVVIFSATGKLFDY

SSSSTKNVVERYKAHTNGVEKSDELSVELQLEIENQIRLNKELAEKSRQLRQMRGEDLEE

LNIDELQKLEQLVEASLGRVIETKEELIMSEIMALERKGAELVEANNQLRQRMVMLSRGN

IGPGLTEPERFINNIGDGGEEGMSSESATNATISSCSSGLSLSLEDDCSDVTLALKLGLP*

>PmDAM3

MMRKKIKIKKIDCLPARQVTFSKRRRGIFKKAAELSVLCESKVAVVIFSATGKLFDYSSS

SIKDVIESYKAHKNGVKKSDEPSVELQLENENHIGLSKELEEKSHQLRQMKAEDLDELNF

DELQKLEQLVDTSLSRVIETKEELRMSEIMALERKGAELVEANNQLKQTMVMLSGGNTGP

TLMDPERLNDNVGGGGEEEGMSSESAISTTCNSALSLSIGDDSDDVTLSLELGLP*

>PmDAM4

MMRKKIKIKKIDYLPARQVTFSKRRRGIFKKAAELSVLCESEVAVVIFSATGKLFDYSSS

SIKDVIERYKARTNGVEKSDEQSLELQLENENRIKLSTELEEKNRQLRRMKGEDLEELDL

DELLKLEQLVEATLVRVMETKEELIMSDIVALDKKGTELVEGNNQMVMLRDRMVMLSKRSTGPALMEPSDSATSTSCNSALSLSLEDECSDDAILSLELGRS*

>PmDAM5

MMNKIKIKKIDYLPARQVTFSKRRRGLFKKAAELSVLCESEVAVVIFSATGKLFDYSSSS

TKDVIERYNADMNGVEKSNNQEIELQLENENHIKLSKELEKTSHQLRQMKGEDLEGLNLDELLKLEQLVEASLGRVMETKEELIKSEIMELERKGAELVEANSQLRQTMVMLSGGNTGPALMDPERLNNNIEGGGEEEGMSAESAISTTCNSAVSLSLEDDSSDEVTLSLKLGR*

>PmDAM6

MVKMMREKIKIKKIDYLPARQVTFSKRRRGLFKKAAELSVLCESEVAVVIFSATDKLFHY

SSSSTEDVIERYKAHTGGAEKSDKQFLELQLENENNIKLSKELEEKSRQLRQMKGEDLEG

LNLDELLKLEQVVEASLGRVIETKEELIMSAIMALEKKGAELVETNNQLRHRMVMLSGGN

TGPAFVEPETLITNVGGGGREDDMSSESAVIATSTSCNSAFSLSLEDDCSDVTLSLKLGL

P*

>PpDAM1

MKMTREKIKIKKIDNLPARQVTFSKRRRGIFKKAAELSVLCESEVAVVIFSATGKLFDYS

SSSMKDVIERYQEHINGAEKFDEPSIELQPEKENHIRLSKELEEKSRQLRQMKGEDLEEL

NFDELQKLEQLVDASLGRVIETKDELIMSEIMALKRKRAELVEANKQLRQRASNYHNHMLSRGNIGPALMEPERLNNNIGGGGEEEGMSSESATSTTCNSAPSLSLEDDSDDVTLSLKLG

LP

>PpDAM2

MVKTMRKKIKIKKIDYLPARQVTFSKRRRGIFKKAEELSVLCESEVAVVIFSATGKLFDY

SSSSTKDVVERYQAHTNGVEKSDEPSVELQLEIENHIRLTKELEEKSRQLRQIKGEDLEE

LNFDELQKLEQLVDASLGRVIETEEELIMSEIMALERKGAELVEANNQLRQRMVMLSRGN

IGPAPTEPERFVNNIGGGGEEGMSSESATNATISSCSSGPSLSLEDDCSDVTLALKLGLP

>PpDAM3

MVKMMRKKIKIKKIDYLPARQVTFSKRRRGIFKKAAELSVLCESKVAVVIFSATGKLFDY

SSSSIKDVIERYKAHTNGVEKSDKPSVELQLENENQIGLSKELKEKSHQLRQMKAEDLEE

LNFDELQKLEQLVDASLGRVIETKEELRMSEIMALERKGAELVEANNQLRQTMMLSGGNTGPTLMEPERLSNNIGGGGEEEGMSSESAISTTCNSALSLSPSLGDDSDDVTLSLKLGLS

>PpDAM4

MVKMMREKIKIKKIDYLPARQVTFSKRRRGIFKKAAELSVLCESEVAVVIFSATGKLFDY

SSSSIKDVIERYEVRTNGVEKSDEQSLELQLENENHTKLSTELEEKNRQLRQMKGEDLEE

LDLDELLKLEQLVEATLVRVMETKEELIMSDIVALEKKGTELVEANNQMVMLRERMVMLSKRNTGPALMEPSESATSTSCNSALSLSLEDDCSDDVVLSLKLGLTVRAGR

>PpDAM5

MMRNKIKIKKIDYLPARQVTFSKRRRGLFKKAAELSVLCESEVAVVIFSATGKLLDYSSS

STKDVIERYNADINGVEKLNNQEIELQLENENHIKLSKELEEKSRQLRQMKGEDLEGLNL

DELLKLEQLVEASLGRVMETKEELIKSEIMALERKGTELVEANNQLRQTMVMLSGGNTGP

ALMDPERLNNNIEGGGEEEGMSAESAISTTCNSAVSLSLEDDSSDEVTLSLKLGR

>PpDAM6

MMREKIKIKKIDYLPARQVTFSKRRRGLFKKAAELSVLCESEVAVIIFSATDKLFDYSSS

STEDVIERYKAHTNDLEKSNKQFLELQLENENHIKLSKELEEKSRQLRQMKGEDLQGLNM

DELLKLEQLVEASLGRVIETKEELIMSEIMALEKKGAELVEANNQLRQKMAMLSGGNTGP

AFVEPETLITNVGGGGEEDGMSSESAIIATSTSCNSAHSLSLEDDCSDVTLSLKLGLP

>PpyDAM1

MVKRMKEKIKIRRIDYLPARQVTFSKRSRGILKKAEELSILCEAEVAVIIFSQTGKLFDY

SSSSTKDVIARYKSHTGGEKWDQITLHQLQLEKENTIRLGKELEDKTRKLRQMKGEDLQD

LDLDQLNKLEKLVKASIGRVIKTKEKKIMSEIMEHANKGAELIKANNQLKQRMVMLSAGGDIGPAGIMELDNLNNVGEEGVTSESATNVTTCSTSAFSLEDDCSDILSLKLGLP

>PpyDAM2

MKIKIRKIDYLPARQVTFSKRRRGIFKKAGELSILCESEVAVIIFSQTGKLFDFSSSSTK

DVIARYNSHVGGEKSDQPTLHQLQLEKENNIRLSKELEDKSCKLRQMKGVDLEDLDLDELQKLEKLVEASLGRVIQTKEEKITSDVMALEKKGAELIEANNQLSQKMVMLPGGDSGPEAILNNIGEESVTSESATNVTTFSNSSLSLEDDCSDTLSLKLGLP

>PpyDAM3

MVKRMNEKIKIRRIDYLPARQVTFSKRRRGIFKKAEELSILCESEVAVIIFSQTGKLFDY

SSSSTKDVIARYKLHTGGEKSDQITLHQLQSEKENTIRLSKELEDKTRKLRQMKGEDLQD

LDLYQLNKLEKLVEASVGRVIKTKEKKIMSEIMALTNKGAELIEANNQLKQRLVMLSARG

DIEPAAIMELENLNNVGEEGMTSESATNVTACSSSALSLEDDCSDILSLKLGLP

>PpyDAM4

MVERMKEKIKIRRIDYLPARQVTFSKRSRGILKKAEELSILCEAEVAVIIFSQTGKLFDY

SSSSTKDVIARYKSHTGGEKWDQITLHQLQLEKENTMRLSKELEDKTRKLRQMKGEDLQDLDLDQLNKLEKLVEASIGRVIKTKKKKIMSEIMAHANKGAELIDANNQLKQRVVMLSAGGDIGPAGIMELDNLNNVGEEGVTSESATNVTTCSSSAFSLEDDCSDILSLKLGLP

>PpyDAM5

MVKRMNEKIKIRRIDYLPARQVTFSKRRRGIFKKAEELSILCESEVAVIIFSQTGKLFDY

SSSSTKDVIARYKLHTGGEKSDQITLHQLQSEKENTIRLSKELEDKTRKLRQMKGEDLQD

LDLYQLNKLEKLVEASVGRVIKTKEKKIMSEIMALTNKGAELIEANNQLKQRLVMLSARG

DIEPAAIMELENLNNVGEEGMTSESATNVTACSSSALSLEDDCSDILSLKLGLP

>PpyDAM6

MKIKIRKIDYLPARQVTFSKRRRGIFKKAGELSILCESEVAVIIFSQTGKLFDFSSSSTK

DVIARYNSHVGGEKSDQPTLHQLLLEKENNIRLSKELEDKSCKLRQMKGVDLEDLDLGEL

QKLEKLVEASLGRVIQTKEEKITSEVMALEKKGAELIEANNQLSQKMVMLPGGDSGPEAI

LELENLNNIGEGSVTSESATNVTTFSNSSLSLEDDCSDTLSLKLGLP

>PpsDAM3

MVKMMRKKIKIKKIDSLPARQVTFSKRRRGIFKKAAELSVLCESKVAVVIFSATGKLFDY

SSSSTKDVIERYKAHTNGVEKSDKPSVELQLENENHIGLSNELEEKSHQLRQMKAEDLEE

LNFDELQQLEQLVDASLGRVIETKEELRMSEIMALERKGAELVEANNQLRQTVSNHHNHMVMLSGGNTGPELMEPERLNNNIGGGGEEEGMSTESAISTTCNSAHSLGDDSGNVILSLKLGLP

>PpsDAM4

MVKMKREKIKIKKIDYLPARQVTFSKRRRGIFKKAAELSVLCESEVAVVIFSPTGKLFDY

SSSSVKDVIKRYKARANGVEKSEESLELQLEHENRIKLSKELEEKNSQLRKMKGEDLEEL

DLDELLKLEKLVEATLVRVMETKEELIMSDIMVLEKKGTALVEANNQMVMLKERMVMLSKRNTGPELMEPSESATSTSCNSALSLSLEDDCSDDVILSLKLGRP

>PpsDAM5

MVRNKIKIKKIDYLPARQVTFSKRRRGLFKKAAELSVLCESEVAVVIFSATGKLFDYSSS

STKDVIERYNVHMNDVDKLNDQEIELQLENENHIKLSKELEEKSRQLRQMKGDDLEGLNLDELLKLEQLVEASLGRVMETKEELIKSEIMELERKGAELVEANNQLRQTMVMLTGGNTGPALMDPERLNNNIGGGGEEEGMSAESAISTTCNSAVSLSLEDDSSDEVTLSLKLGR

>PpsDAM6

MVKMMREKIKIKKIDYLPARQVTFSKRRRGLFKKAAELSVLCESEVAVVIFSATGKLFDY

SSSSIEDVIERYKAHTNGVQKSNKQFLELQLENENHIKLSKELEEKSRQLRQMKGEDLEG

LNLDELLKLEQLVEGSLGRVIETKEELIMSEIIALEKKGAELVETNNQLRQRMAMLSGGN

TGPALVEPETLNTNIGGGGEDGMSSESATMATSTSCNSALSLSLEDDCSDVTLSLKLGLP

>MdDAM1

MAREKIQIKKIDNATARQVTFSKRRRGLFKKAEELSVLCDADIALIIFSSTGKLFEYASS

SMKEILERHNLHSKNLDKLEQPSLELQLVENSNYSRLSKEIAAKSHQLRQMRGEEIQGLS

LEELQQLEKSLEAGLGGVVEKKSEKIMKEINDLQRNMNVQAMQLTEENERLRQQVVEKSNGRRLVHVDSENLITEEGQSSESVTNLCKSNSGPQDYDNSVTSLKLGCA

>MdDAM2

MVKIRKEKIKIRRIDYLPARQVTFSKRRRGIFKKAEELSILCESEVAVIIFSQTGKLFDF

SSSSWKRDIIHVNLIKLWSSPPDNQGELTSFPMFGIGQGHRNTKDVIARYKSHTGEKSNQ

SMLDQLQLEKENTIRLSKELEDKTRKLRHLKGEELQDLDLDELQKLEKLVEASHGRVMET

KGDELVEANNQLKQRMVMLSARGDIGPAAIMELENLNNGGEEGVTSESATNVTTSSNSPL

SLEDDCSDILSLKLGCAALTS

>MdDAM3

MKIKIKKIDYLPARQVTFSKRRRGIFKKAGELSILCESEVAVIIFSQTGKLFDFSSSRLW

SSPPXNQDELVSFPFVDSHAIESDKLIMHVNRRTKDVIARYNSHIGGEKSDQPTIHQLQL

EKENNIRLRKELEDKSCKLRQMKGVDLEDLDLDELQKLEKLVEASLGRVIQTKGAELIEA

NNQLSHRMVMYPRGDIGPEAILELENLNNIGEESXTSESTTNVTTCSNSSLSLEDDCSDI

LSLKLG

>CsDAM1

MVRQRIQIKKIDNVTSRQVTFSKRRKGLFKKAQELSTLCDAEIALIVFSATGKLFEFSSS

SMRQVIERHNLESGNLVNLNQPSLEQQLENSGCTILSKEVNKKIHELRQLRGEELQGLDA

EELKNLEKSLEGGLSRVLKTKGEIMEKEITARERKEARLVEENVWLKQKVPMEIVKIGQT

HDDQQGQSAEFITNNGSSAAPPQDNDSSDTSLKLGLPFPELN

>CsDAM2

MVRQRIQIKKIDNVTARQVTFSKRRRGLFKKAHELSTLCDAEIALIVFSATGRLFEYASS

STRQVIERHNLQPQNLVQLNQPSLELQLENSTRAMLSKEAEERTLELRQLRGEELHELGF

EELKKLEKSLEGGLSRVLKTKDDRVEKEIAALRRKEARLMEENAWSKQQVQMQIVNMGQPQEQGQSSESITNNGSTVAPPQDYDSSDTSLKLGLPYQS

>PmSVP1

MAREKIQIKKIDNATARQVTFSKRRRGLFKKAQELSVLCDADIALIIFSSTGKLFEYASS

STKEILERHNLHAKNLSKIEQPSLELQLVENSNYSALSKEITAQSQQLRQIRGEEIQGLN

LEELQQLEKSLEAGLGRVIEKKGEKIMKEISDLESNAMRLVEENERLRQQVLEKHNSQRP

VRADSENMVMEEGQSSESVTTNLCNSNSAPQDYESSDTSLKLGLPYSG*

>PmSVP2

MTRRKIQIKKIDNTTARQVTFSKRRRGLFKKAQELSTLCDAEIALVVFSATGKLFEFTSS

SVQQVIERHHLLSSDFDKLNHPSLELQSFCMSPLESSTSAALSKEIAEKTHELRKLRGEE

LQELNMKELQELEKLLGSGLRRVRDAKCEIVLKEITSLKWKGSQLMQENKRLKQMANRQVQTLELEQGQSSEPIGNFIHSNPSQDHDSSDTFLKLGLAFPSGI*

>PpSVP1

MEIEAAEEAEEAEEAEAEEEDRSMAREKIQIKKIDNATARQVTFSKRRRGLFKKAQELSV

LCDADIALIIFSSTGKLFEYASSSMKEILERHNLHAKNLSKIEQPSLELQLVENSNYSAL

SKEITAQSQQLRQIRGEEIQGLNLEELQQLEKSLEAGLGRVIEKKGEKIMKEISDLESNA

MRLVEENERLRQQVLEKHNSQKPVRADSENIVMEEGQSSESVTTNLCNSNSAPQDYESSD

TSLKLGLPYSG*

>PpSVP2

MTRRKIQIKKIDNTTARQVTFSKRRRGLFKKAQELSTLCDAEIALVVFSATGKLFEYTSS

SVQQVIERHGLLSSNYDQLNQPSLELQSFGMSQLESSTSAALSKEIAESTHELRKLMGEE

LQELNMKELQELEKLLGSGLRRVRDAKGEFFLKEITSLKWKGSQMMQENKRLKQMANRQVQTLELEQGQSSEPIGDFIHSYPSQDHDSSDTSLKLGQAFPNGI*

>PpySVP

MAREKIQIKKIDNATARQVTLSKRRRGLFKKAEELSVLCDADIALIIFSSTGKLFEYASA

SMEEILERHNLHSKNLDKLEQPSLELQLVENSNYSRLSKEIAAKSHQLRQMRGEEIQGLS

LEELQQLEKLLEAGLGGVVEKKSEKIMKEINDLQRNAMQLTEENERLRQQVVEKSNGRRLVHVDSENLITEEGQSSESVTNLCKSNSGPQDYDSSVTSLKLGLPYSG*

>PpsSVP

MTRRKIQIKKIDNTTARQVTFSKRRRGLFKKAQELSTLCDAEIALVVFSATGKLFEYTSS

SVQQVIERHGLLSSTFDKLNQPSLDLQLESSTSAALSKEIAENTHELRKLMGEELQELNM

KELQELEKLLGSGLKRVRDAKGEIVLKEITSLKWKGSLLMQENKQLKQMANQQIQTLELELEQGQSSEPIGDFIHSDPSQDHDSSGTSLKLGLAFPNGI

>MdSVP

MAREKIQIKKIDNATARQVTFSKRRRGLLKKAEELSVLCDADIALIIFSSTGKLFEYASS

SMKEILERHNLHSKNLEKLEQPSLQLQLVENSNYTRLSKEIAAKSHQLRQMRGEEIQGLN

LEELQQLEKSLETGLGRVIEKKSEKIMKEIGDLQRNGMQLMEENERLRQQVAEKSDGRRL

VQVDSENMFTEEGQSSESVTNPCNSNNGPQDYDSSDTSLKLGCV*

>CsSVP1

MGVFLIAHYYLSIYIYIFEERERLLYIEMAREKIQIKKIDNVTARQVTFSKRRKGLFKKA

EELSVLCDADVALIIFSATGKLFEFSSSSMTEILERRNLHSKNLDKLDQPSLELQLVENS

NFSRLSKEVAEKSHQLRQMRGEELPGLSIEELQQLERSLEAGLSHVIGKKGEKIMKEISN

LQQKEMELMEENERLRQQVKEVSNARKHVAVATDSENMYINEEGQSSESVNICNSTSPPQ

DYDSSVTSLKLGLPYSG

>CsSVP2

MVRQRIQIKKIDNVTARQVTFSKRRRGLFKKAHELSTLCDAEIALIVFSATGRLFEYASS

STRQVIERHNLQPQNLVQLNQPSLELQLENSTRAMLSKEAEERTLELRQLRGEELHELGF

EELKKLEKSLEGGLSRVLKTKDDRVEKEIAALRRKEARLMEENAWLKQQVQMQIVNMGQPQEQGQSSESITNNGSTVAPPQDYDSSDTSLKLGLPYQS

>PmMADS03

MSEGSIEFILLNVGLQIKMGRGKIEIKRIENTTNRQVTFCKRRNGLLKKAYELSILCDAEVALIVFSSRGRLYEYSNNNIRNTIERYKKACSDSSGSTSITEINAQYYQQESAKLRQQIQMLQNSNRHLMGDALSTLSVKELKQLENRLERGINRIRSKKHEMLLAEIEYLQKKEIELENENVCLRTKISEVERLQQANMVGPELNAIQALASRNFFSQTMMEGGATYPQQDKKILHLG*

>PmMADS15

MAYENKSMSLDSPQRKLGRGKIEIKRIENTTNRQVTFCKRRNGLLKKAYELSVLCDAEVALIVFSNRGRLYEYANNSVKETIERYKKACAESTNTGSVSEASTQYYQQEAAKLRAQIGNLQNSSRHMMGESLSSMNMKDLKNLESKLEKGINRIRSKKNELLFAEIEYMQKREIDLHNNNQLLRAKIAENERSQQNINVMAGGGSYEIMQSQPYDSRNYFQVNALQPNHQYNSRQDPMALQLV*

>PmMADS12

MGRGKIEIKRIENSSNRQVTYSKRRNGIIKKAKEITVLCDAKVSLVIFASSGKMVEYCSPSVTVTDILDKYHGQAGKKLWDAKHENLSNEVDRVKKDNDSMQVELRHLKGEDITSLTHKELMALEDALENGLASIRDKKSKFVDILRENERALEEEHKRLTYELHKQEMKIEENVRELENGYHQRLGNYNNQIPFAFRVQPIQPNLQERM*

>PmMADS13

MTRGKIQIKRIENATNRQVTYSKRRNGLFKKAHELTVLCDATVSLIMVSSSGKIHEYISPSTTTKQFFDQFQKTKGVDIWSSHYEVSHSSLLSLSLSLSLSLSIYIYMYIIVWLCVQAMQEHLKKLKEVNRSLQKQIRQRVLGECLNDMSFDELRGVEQEMEGAVDVIRKRKVDARDDTHYRLVENGGEDYESAFGYSSNGGPRIFA*

>PmMADS14

MGRGRVELKRIENKINRQVTFAKRRNGLLKKAYELSVLCEAEVALIIFSNRGKLYEFCSSSSMLKTLERYQKCNYGAPETNVSAREALELSSQQEYLKLKARYEALQRNQRNLLGEDLGPLSSKELESLERQLDMSLKQIRSTRTQCMLDQLTDLQRKEHMLNEANKTLKQRLFEGYHVNSLQMNPNADEYGRQQAQAHGDGFFHPLDCEPTLQIGYQNDPISVVTAGPSVSNYMAGWLP*

>PmMADS17

MGRGRVELKRIENKINRQVTFAKRRNGLLKKAYELSILCDAEVALIIFSNRGKLYEFCSSSSSILKTLERYQKCSYGQVEVNKPAKELEQSSYREYLKLKGRFESLQRTQRNLLGEELGPLNTKELEQLERQLESSLKQVRSTKTQYMLDQLSDLQNKEQMLIEANRDLSLKLDDISSRNQIRQSWEGGNQGGMAYGTQHAQSQGFFQPLDCNPTLQIGYSNVGSEQMSATTHAQQVNGFIPGWML*

>PmMADS18

MGRGKVQLKRIENKISRQVTFSKRRAGLLKKAHEISVLCDADVALIVFSTKGKLFEYSSDSSMESILERYDQYSHAEQQLTTDFDPQGSCWSLEYPKLAARIEVLQRKLRHFTGEDLDSLSLRELQNLELQLETALKRIRTRKNQLMHESISVLHKKQKALQEQNNSLGKKLKEKENMLEVEHDGQVQQVEQHQSNQAAHNSSTLMLMPPPPPQTSSTPALLASLTIGGGIQARGGMEDGDDNDGRTQTRPPPTTNTLMPMWMYRHFNE*

>PmMADS20

MGRGRVELKRIENKINRQVTFSKRRNGLLKKAYELSVLCDAEVALIIFSSRGKLHEFGSAGFIWILSLPIKTSNEAVASQVLEAPEMKSCSSYLLSSRSMQSWYQEVTKLKAKYESLLRTQRQLLGEDLGPLNVKELQNLEKQLEGALAQARQRKTQLMIEQMEDLRKKERHLGDLNKQLRVKLETEGQNLKAIQNMWSSNAAAGSSSFSFHSSQTNPMDCQPHEPVLQIGYHQYLPVEGPSMSKSMACETNFIQGWVL*

>PmMADS21

MVRGKTQMRRIENATSRQVTFSKRRSGLLKKAFELSVLCDAEVALIIFSPRGKLYEFASSSMQTTIERYQKHTKDNLTNNKSVSTDQNMQHLKQESSSMMKQIELLEVSKRKLLGEGLGSCSIEELQEIEQQLERSVSNVRARKTQVFKEQIEQLREKGKALAAENEKLIEKCGRIQPRQASNEQRENLAYTESSPSSDVETELFIGLPERRKR*

>PmMADS22

MVRGKIEMKRIENATSRQVTFSKRRNGLLKKAFELSVLCDAQVSVIIFSQKGRLYEFSSSDMQETIKRYHKHAKAGQTNKIEVEEYVEQLKHESTAMAKKIENLEASQRKLLGHGLDSCSVEELQEITGQLERSVRKIRERKAHLFAEQMEQLRAKERLLLEENAKLSEEFGAQPRLLLQQQQLSVEEKGAVSYWSLSSPSSEVETELFIGPPVTRC*

>PmMADS23

MGRRKVVLERIENKINRQVTFSKRRNGLLKKAYELSVLCDAQVALIISSSRGKLYEFGSTDYNSVHSLISVNKILERYRQCCYSLQGNVAENETQNLYQEVSKLKVKYESLQLSQRHLLGEDLEKLRLKELVNLENQLDKTLSKARQRKVLVICVM*

>PmMADS25

MVRGKTQMKRIENAASRQVTFSKRRNGLLKKAFELSVLCDAEVALIIFSTRGKLYEFSSSRRFSIGNTLDRYQKRVKDQGLGSKAVQVDMEHGKDDTSSMAKKIDFIEASKQKLLGNCLESCSIEELQQTENQLERSLSKIRARKTQLLREQIENLKEEEKNLFEQNAKLREKCGMQPLGPPSARKDEENCAVRQPRTPDMEDVETDLVIGPPERRRSSQNP*

>PmMADS27

MGRGRVQLKRIENKINRQVTFSKRRSGLLKKAQEISVLCDAEVALIVFSTKGKLFEYSTDSCMERILERYERYSYSEKQLLANDNESTGSWTLEHAKLKARVEVLQRNHSHFMGEDLQSLSLKELQNLEQQLDSALKHIRSRKNQVMYESISELQKKDKALQEQNNLLAKKVKEKEKALAPQAQSWEQQVQNQGLDCSSTLLPEALHSGSNYQGIRSDGFGGDHEDENETPTANRPNTLLPPWMLRHLNE*

>PmMADS28

MGRGRVELKRIENKINRQVTFAKRRNGLLKKAYELSVLCDAEVALIIFSSRGKLYEFCSSMSMLKTLEKYQRCSYGSLEANRPVNDTQKQNSYQEYLKLKARVEVLQQSQRNLLGEDLAPLNTKELEQLEHQLEASLNQIRSTKTQFMLDQLCDLQNKEQMLVEANKALRRKLEETSGQAPPPLAWEAAGHGNNNDQHTRLPHHPHSQGFFHPLGNNSTSQIGYTPLGSDHHEQMNVGNHGQHVNGFIPGWML*

>PmMADS29

MGRGKIEIKLIENHTNRQVTYSKRRNGIFKKAQELTVLCDAKVSLIMLSNTGKMHETKRMYDDYQKTLGVDLWSSHYQAMKDTLWKLKEINNKLRREIRQRLGHDLNGLTYEQLHSLEDKMASSLEAIRERKYHVLKTQMETYKKKVKNLQERRGNMLHGYFDQEVASEDPQYGYVDNEGDYESAVALANGASNLFTIHLHQDIRDHANLHHHGGSSLGSSITHLHDLRLA*

>PmMADS31

MGRGRVQLKRIENKINRQVTFSKRRTGLLKKAHEISVLCDAQVALVVFSNKGKLFEYATDSCMDQILDRYERYSYAERQLVEPDFESQCNWTFEYSRLKAKVELLQRNQRHYLGEDLDSLTLKEIQSLEHQLETALKQIRLRKNQLMHESISELQRKERAMQEQNNLLAKKIKEKEKAAAEEVHNWEQQNNGLNMLPQPLPCLNMGGTQQDEFLQVRRNQLDLTLEPLYSCNLGCFAA*

>PmMADS32

MGRGKVELKRIENKINQQVTFAKRRNGLLKKAYELSVLCDAEVALIVFSTRGKLYEFCSGSSMEKTLERYQRCSYSALEASQPAQDSQSRYQDYVNLKAKVEVLQLTQRNFLGEDLGHLGTKELQQLENQLDMSLRQIRSTKTQVMHGQISDLLRKEQMLLEANNELRRKLEECDAAIERYSWTTEEQNQNVPNSSHHQAAQFEGVLDHSQCNNTLQIGYNPAAVTDHHELQSSTQSHSGLIFPGTWVL*

**Supplementary Data4.** The genome sequences of SVP1 and SVP2 promoters, 2000 bp up stream fragments.

> Pro-PmSVP1 (Pm002166)

GGACATTCTTCGTTGTTTGCGGGCACAAATCAAACTCGCAACTCACTTTAGCCACATTCTACTATGTGAATGTCAAATACTGTGACCTCAAAAGACGAGAAAGACATTGATGAATGGAGAAAATGCTTTAGAATGATTCTAATTAGGGACATATCGCATATGATAAATTATATATCTTCAAGATGAGATGAGGGAGCGTGGGGACTACCATGACAGAAACACTTGCACATCAACAATTTTAACCATTTTTACCACTTGGTTTAGTTCTATTTGTGTCTATCTAGTTGGAGGAGGTTTTAAGTTTGACTCACATGACATAATTTGTGTGTGTGTGAGAAATCTCATTTCCCTTTAGTTTAGACCATGACTTATACTAAGAAAATATACATCCAAATTGAGCTCAATGCTTCCCCTGCACTGCAAACTCAACATTTCAGTTTTTCACACCATCGGTTAGACCATCTCCAACCCTTGGGGTGGAAACTCAAATTTTGAGTTTAGCCTCCAAAAACCATCTCCAACCCATGTATTTTGGGGGGTGGAAATTTGAGAAATCTCAAATCTTGGGTGGAATTCCAGTCCCATTTTTGCCTGGACTGATAATTTTGTGGGCCCAAAATCTGCTTTTTTTATTATTTTTTTTCTGACTGAAGTCCATGTGCTACACAATGTTTGCTGGGCCCAGTTTTGGTTACACTGTTTGGGCAGCAACTTTGTGCCAAAAATCTGATATTGCTGGTTTGTTTGTTTGTTTTTTTTTTTTGTTTGGTTTTATTTTAAATATTCCAACAACTATATTTGTAATCAATAATCATGATTAATTTTAAGCAATCTCAACGGCAAAATTTAATGATTGTCGTTTATTTAAAAATGCTTAGATAATTTAAACACACTTACATAATTCAATGAAGAGTTCGAATTATTCCGAGCATACGTATAGTATTGTACTTATTTTTATTTTTTAAGTGAAATTCAATTATGTTTTGGAATTTTATTTTTTTTAGGTTAAAATGTTTAGAAAATTAAATCACGAGTGTTGAAACAAAAATAATGTCAAGAAAAATTAACAAGAAACAAAATTTAGGGGTTAATCATTCTTAAACAATTTAATAAAGTTGCGGACTCAAATTATGAGTCTACAAGGTTGGAGGAAAAAAAAATTTGAGTCCTGAAAATACATGAATAGTTGTATTTTGGAGGGTGAAAATTTGAGTTTAGCCCCAACATGGTTGGAGATGGTCTTATACCTTTTTAAAGCAACCAAAGGGCAAAGAAATAGTGACCAAAGGGCGAGGACGTGGAAAACAGCCAGTAGTTTGCTTCACCATAAAAAGGACCTTCCTTTTCCACACTTCCACAAACATTCCCTCTCTCTATAATTTTTTGTCTCTCTCTCCCAATCTCATTGGTGGCCTTGAAGTTTCACAGAACACTAAACCCCAGATTCACTCAGACTGGTGAGTAAATCATTAAATACAAAGCAAATAAAAACTTAAAAAGGCACCAAACAGATCCCCTTTTTACCTAATAATCTAGAAGCCCCTCCCCTAGCTTATCCTGAGAAAATGTTTCTTTTCTCCAATTGGTTGGTTTATTTTTTATTTTCTGAGCCTTGTTGGTGTTGAAAAAGCTCTCTCTGGATCTAAGAATCTAGAGATGGAGATGGGTCTCTTTCTCTCTAAAATTTTGCATTTCATTTCATTGCATATTTAGGGTTTGCCTTTGCCCCCAGAGATTCTTGGCTAAATTTGGCAAAATGCCGCCACTTCTCGCACCAGACCTGTCCCTCTCCAATTAGCCGTCGTTTCCTTTTGCTTCTAATTTCTTTGCCTTTTCTGGTTAGTTTTTTGCTGATTATATTCTCTGACTGAAGATAACTAAACCCTAAAGAAGAAAGCAAAATATTCCATTCCAAACAAAGTTAAGAGTTTTCTTTCTATAATATATCATTCTCCTTTTTTCACAAGCTTGCAGAAGAAGCAGAAGCAGAAGATCGATCG

> Pro-PmSVP (Pm022002)

TTTTTCAAAGTGTTTTGGTCGGTTGTTTATGCTGACTTGATCATAAAGGGTGCGGCTGGTGAGTTAAAACATCGGGTCGATCATCAACTTGGTCAGGTATGATGGGACTAGATGGCGAGGTCTTTGCTGAAGTGTTGTCTATGTGGCATCATGTTAATGGTCAATTGGCTCGGATCAAATTATGTCTCAACAATAGGGGTGAAATTTACAAGTTAATCCAAACTAGGGCCTCTTGTCGGACAATGTGACATAGTTTGAATCACATGTTTAATTAAGAACTTATTTCATTGATTATGTGTGTAGCTTTTTCATGAATTTGTGTATGAGTTTGTAGAGTCTTATAAGTTCGCGTACCAGTTTGAAATGACCTGTAAAATTTAGGAGGGTTTTCATGTAGTAAACTCGTAATAAAAATTACATAATGACCCAAAACATATTGTAAGAAGTTTGACAAAAATAAGAAAAGAATATGTGTAAGAAAAGAAAACCATCCTAATCCCCTCCATTGATGTGACGTATGTTCCCTTTTCCACCCTACCAAATAAAAACTTACATATATATATATATATATTGGAATATTACTGTAGTGATATTTCAAGTCAATGACACATTGTTCTCCATTTTAATTAATTTTTTCATATGATTGTGTTATTGACTTGAGATATTGTTATACTTCTCCGTTGCATTACCTCTATTGAGTGATTGAGTGACTGAGATGATGCCCATTATTTAAGCATTAGCTAATATCCCAATTAAAAACACTAATTTTGAAGGTGAAGATATGAACAGTGAATCCTTCACATCCCGAGAATTTGAACGGTAGGAAGCATGGCATGGTACGAGGGTACAACCATGCCATCATTCTTGCTCTGTCATACGACATGACTCTCTGTACGGGAAAGCAAACCTCACCCACATCAACTATAAAAATAAAATAATAATTTCACTGTATTAGTTAGGCGAAAGACCAACCACACAGAGAGTCCACTACTGTCCATTTTCCATTAAATTCCCTTTACCTCTTTTAAATAAGAAAAAGTAAATAAATATCATCTGAGGGAGATATACCGAAATTGCAGAGATGAGAGAACCTTTTGTTTGGCTTTAAAAGGAATCTTAACTTTTCTTGAACTAGGTCACTCTCTCTCTCTTTCTCTCTCTCTCTCTCTCTCTAGATCATTGGTGACTTTCTTTCTACCTGGCTCTTGTGGGAAGTGTCAACGACACTAAATACGACGTATGTAAAGAAAAGAGGTAAAGAATATTTGAAATGAAATCAGAAGCAAAAATATATAAACGTTTTGTGAGAATCTCACTGGAATTTGCAGACACAATGAGTTAATTGGATTGCTAGGGTTGTTTCTCTCCTTCTGTTTCCCTCTTGGGTTTTCTAAGATTTTCTTCTTTGAATCTAGCTAGGGTTAGTTTTGAAAGGTGCAGCTGGCTCTAACACCAATTGATTCTTTTTTATGTTCTCCTTTTGCTCTTACCCAAAACCTATGATCTTTTTTCTTCTCTCTCCAGATACCATTTCTTCCCACTCTCTAATATTTCCATTTACAGAAGTACCACGCTGTCTCCACTTCGATTGATTTGATTTGCTCTAAATAATATTTTTATATTATTTGTATATTTGTTCGTTTTTTCTTTGTATTTCTGGGTTTAGAGGCAAGTTCTCTTTGATTTTTTTTATTTATGGTTCTTTTGTTTCTTCTATCTCTCTCTTATTTTTCCCATATGTCCAGAAACTCACTTCCTCTTTCTAGTTTTGTTCAAATGGTTTGATTTATATGATGGGGTATCTTTAATTTCCCTTTTTTCATTCCACATTTACTCAGAATAGTTGGATCTGAAACTTCATTAATTTTATTATGCAGTCTCTCTCTCTCTCTCTCTCTCTCTCTCTCTCTCTATGTATATATATATAGTTTGTGTATGATCGTTTGATAATGATGAAGTGATTTTATGTGAAGGGGTTTCTGCATTTTCCTTGTAATTAA
